# Non‐Globular Organic Ionic Plastic Crystal Containing a Crown‐Ether Moiety – Tuning Its Behaviour Using Sodium Salts

**DOI:** 10.1002/cphc.202200258

**Published:** 2022-06-28

**Authors:** Anna Casimiro, Jody Lugger, Johan Lub, Kitty Nijmeijer

**Affiliations:** ^1^ Membrane Materials and Processes Department of Chemical Engineering and Chemistry Eindhoven University of Technology P.O. Box 513 5600 MB Eindhoven The Netherlands; ^2^ Stimuli-responsive Functional Materials and Devices Department of Chemical Engineering and Chemistry Eindhoven University of Technology P.O. Box 513 5600 MB Eindhoven The Netherlands

**Keywords:** chaotropic anions, crown-ethers, organic ionic plastic crystals, sodium salts, soft materials

## Abstract

Organic ionic plastic crystals (OIPCs) are a class of soft materials showing positional order while still allowing orientational freedom. Due to their motional freedom in the solid state, they possess plasticity, non‐flammability and high ionic conductivity. OIPC behavior is typically exhibited by ‘simple’ globular molecules allowing molecular rotation, whereas the interactions that govern the formation of OIPC phases in complex non‐globular molecules are less understood. To better understand these interactions, a new family of non‐globular OIPCs containing a 15‐crown‐5 ether moiety was synthetized and characterized. The 15C5BA molecule prepared does not exhibit the sought‐after behavior because of its non‐globular nature and strong intermolecular H‐bonds that restrict orientational motion. However, the OIPC behavior was successfully obtained through complexation of the crown‐ether moiety with sodium salts containing chaotropic anions. Those anions weaken the interactions between the molecules, allowing rotational freedom and tuning of the thermal and morphological properties of the OIPC.

## Graphical abstract

**A new family** of non‐globular organic ionic plastic crystals (OIPCs) containing a 15‐crown‐5 ether moiety was synthetized and characterized. The initial 15C5BA molecule did not exhibit the desired behavior because of its non‐globular nature and the strong intermolecular hydrogen bonds, which restrict orientational motion. However, OIPC behavior was successfully achieved through complexation of the crown‐ether moiety with sodium salts containing chaotropic anions.

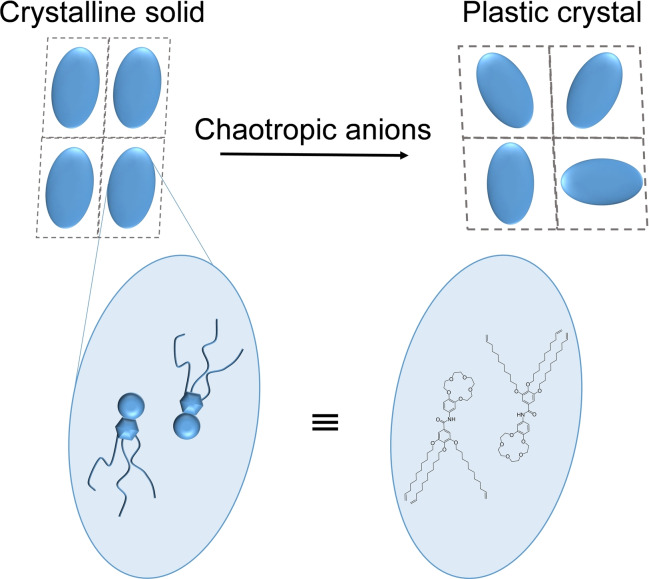

## Introduction

**Figure 1:**
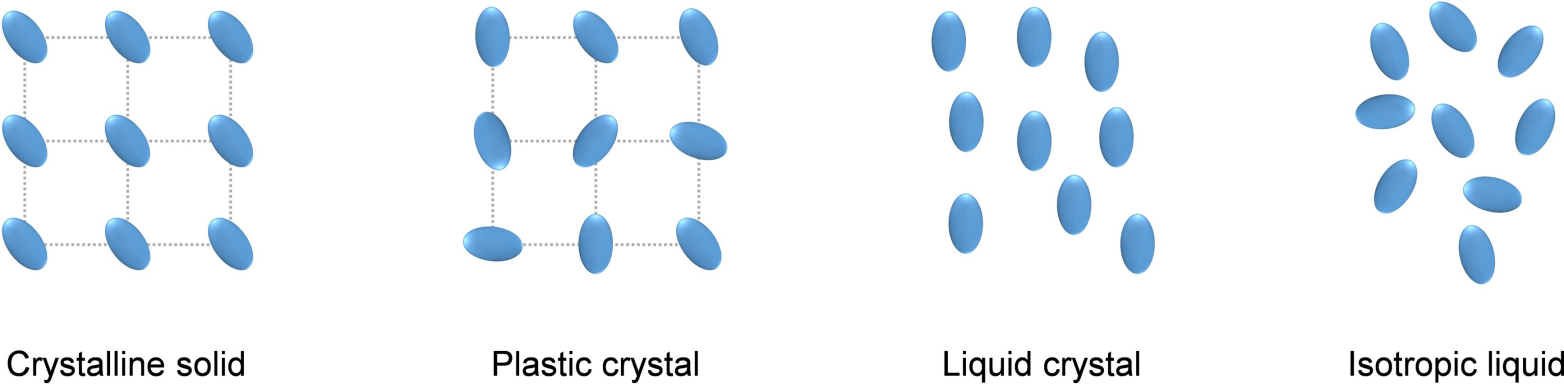
Representation of states of matter from crystalline solids to isotropic liquids.

Plastic crystals (PCs) are a class of soft materials that, similarly to liquid crystals (LCs), can be considered as a phase in between crystalline solids and isotropic liquids (Figure 1).

While LCs have orientational order but do not possess long range positional order, plastic crystals show the opposite behavior; arranging in a crystal lattice, like crystalline solids, with long range positional order but with the orientation of the molecules not being fixed, like for isotropic liquids (Figure 1). PCs are typically formed by ‘simple’ neutral organic molecules and, albeit having long‐range positional order, they do show mechanical plasticity.^[1]^ After their initial discovery by Timmermans^[2]^ in 1938, PCs have been studied by various fields, such as ferroelectrics,[^3^, ^4^, ^5^] magnetics[^6^, ^7^] and optics.^[8]^

**Figure 2:**
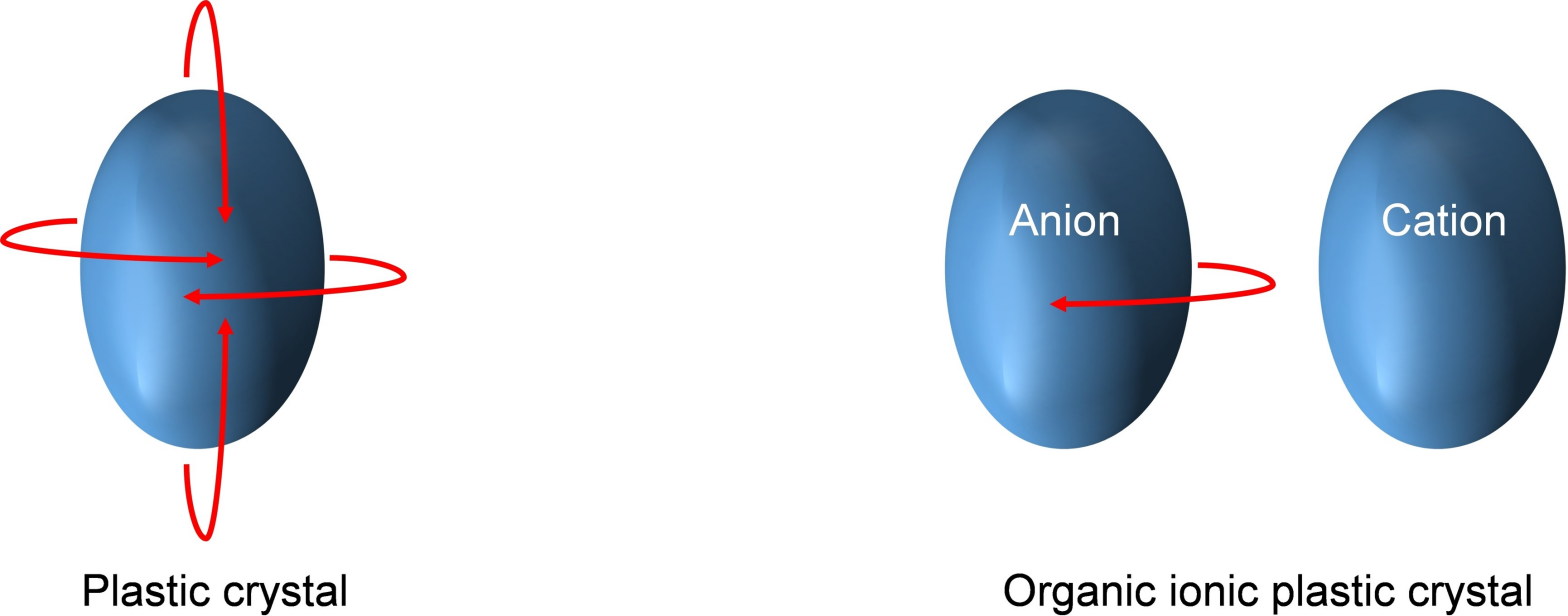
Pictographic representation of the rotator phase in (left) a plastic crystalline system, (right) an organic ionic plastic crystalline system in which only the anion can rotate.

Organic ionic plastic crystals (OIPCs) are considered a sub‐class of plastic crystals, and similarly to PCs, OIPCs possess long‐range order. However, unlike PCs which are constituted by a single non‐charged species, OIPCs are formed by the interaction of two ionic species (Figure 2).

These ionic species form an organic salt and can be considered as the solid counterpart of ionic liquids (ILs).[^9^, ^10^] The attention towards OIPCs is significantly rising due to their plasticity, non‐flammability, low volatility, high ionic conductivity in the solid state and high electrochemical and thermal stability.[^8^, ^10^, ^11^, ^12^] Their low plasticity and low volatility make them easier to process and more sustainable compared to conventional PCs.[^9^, ^10^, ^11^, ^12^] Moreover, some OIPCs can be in their most conductive, plastic phase around ambient temperature.^[13]^ Due to aforementioned characteristics, there is a growing interest in OIPCs as a potential solutions for various electrochemical applications, e. g. as solid state electrolytes in fuel cells and in batteries. For both PCs and OIPCs, the short‐range disordered phase (plastic phase) is typically reached by going through one or more solid‐solid phase transitions (indicated as phase I, II, III etc.) before melting occurs. The phase that precedes the isotropic melt phase is considered the most disordered phase and named phase I. After melting, the material reaches the isotropic state in which it does not possess any positional or orientational order anymore. The solid‐solid phase transitions (III→II, II→I) characterize increasing molecular rotations and for this reason, the plastic phase is also called “rotator phase”[^9^, ^14^, ^15^, ^16^, ^17^] (Figure 2). The introduction of a certain amount of disorder before melting, has an entropic consequence on the melting behavior of those compounds. When PCs or OIPCs melt, the entropy of fusion ΔS_fus_ is very small.^[9]^ In this regard, Timmermans noticed that the materials that showed PC behavior had a ΔS_fus_ <20 J ⋅ K^−1^ ⋅ mol^−1^.^[2]^ This value came to represent a discriminant criterion for the determination of PC phases. However, in recent years it has been noticed that in the case of OIPCs, this entropy of fusion can also be higher.[^9^, ^18^] This is attributed to the fact that in some OIPC systems just one of the two components of the salt exhibits rotator motions. The other component might possess internal degrees of freedom that are destroyed only after melting^[17]^ (Figure 2).

**Figure 3:**
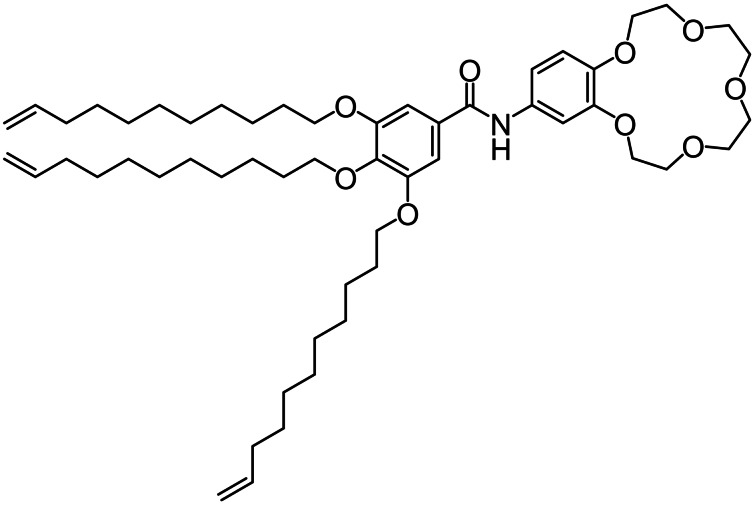
Schematic structure of 15C5BA.

Commonly studied OIPC salts are formed by a combination of pyrrolidinium,^[18]^ imidazolium^[19]^ or tetralkylamonium^[20]^ cations and I^−^, PF_6_^−^, BF_4_^−^ or SCN^−^ as anions. It is interesting to note that, especially with respect to the study of OIPCs, it is well known for the anion choice to have large implications in terms of resulting molecular order, organization, physical and chemical properties of the systems being studied.[^21^, ^22^, ^23^, ^24^, ^25^, ^26^] And in case of OIPCs, either one or even both of the ionic components can have rotational freedom in the PC phase (Figure 2). Rotational freedom, a globular shape and weak intermolecular interactions all contribute when wanting to achieve a significant amount of disorder before melting. Currently, however, a complete understanding of the mechanism of formation of OIPC phases is still unavailable.^[1]^ OIPC systems formed by small, simple molecules have been thoroughly studied.[^2^, ^18^, ^19^, ^20^] The molecules typically have a globular structure, possessing symmetry around their center^[27]^ and allowing for orientational degrees of freedom.^[1]^ In addition, such molecules generally lack strong intermolecular interactions that could potentially disfavor the formation of the rotator phase and concomitant plasticity.[^4^, ^5^, ^27^] However, the growing interest in this class of materials necessitates more in‐depth insights on OIPC behavior, and perhaps especially in case of more complex, non‐globular systems, to widen the scope of OIPCs; ideally to be able to predict, control, and tune OIPC properties towards specific applications and make optimum use of the unique combination of features of this class of materials. In an attempt to address the latter, the current work reports on the design, preparation, and characterization of a new family of OIPCs. However, unlike earlier studies, the herein described base material has a non‐globular structure, and exhibits strong intermolecular interactions. To tune its behavior and morphology we synthesized the monomer. Subsequently we performed its complexation with different sodium salts (potentially forming the OIPC) and then systematically investigate how the nature of the anion determines the order of the non‐globular complexes prepared. The basic molecular structure of the OIPC (hereinafter referred as 15C5BA, Figure 3) was rationally designed to promote intermolecular interactions by means of strong H‐bonds between the amidic functionalities and π‐π stacking between the aromatic rings.

**Figure 4:**
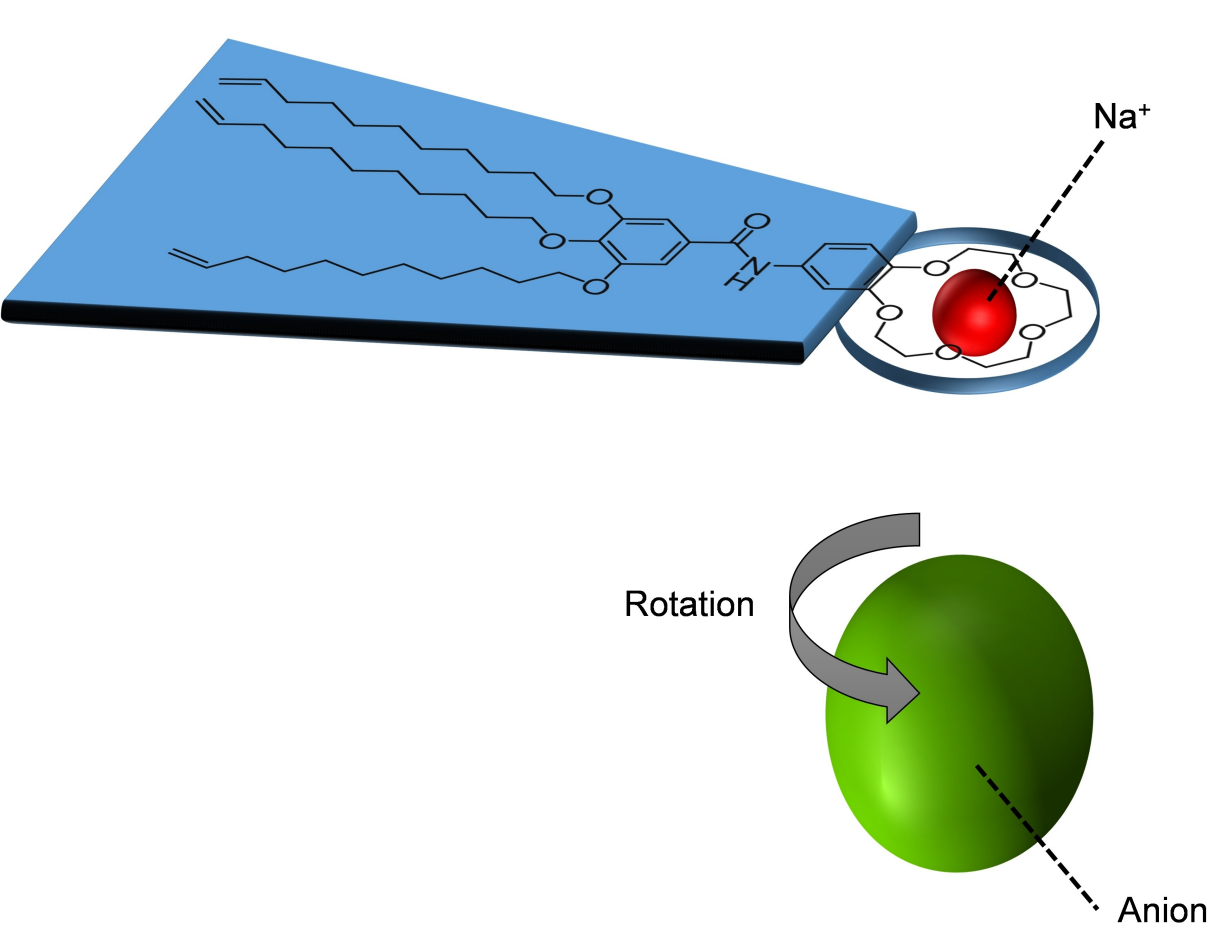
Pictographic representation of the host‐guest interaction between 15C5BA and a sodium salt. Hypothesis on molecular rotation motion.

These strong interactions should allow for the formation of a certain degree of order. Furthermore, to ensure enough flexibility to the molecules and facilitate OIPC formation, three alkyl chains, each containing a double bond, were included in the structure. The double bonds could potentially allow for future polymerization of the system. The presence of the 15‐crown‐5 moiety in the molecular structure allows for the complexation with different sodium salts. This host‐guest interaction between 15‐crown‐5 and Na^+^ is designed to significantly decrease the degrees of freedom of the sodium salts while still allowing for the rotator motion typical of OIPCs (Figure 4).

The counterion of the sodium salt has a key role in the formation of the OPIC phase: it should interact with the H‐bonding sites of the 15C5BA molecule and, by weakening the strong interaction that could prevent molecular rotation,^[28]^ significantly help the formation of the plastic rotator phase^[27]^ regardless of the non‐globularity of the system. Complexation with different sodium salts is investigated through ^1^H NMR, ^13^C NMR and ATR FT‐IR. Moreover, ^1^H NMR and ATR FT‐IR are applied to study the nature and entity of the intermolecular interactions arising between the molecules. To confirm the OIPC nature of the compounds prepared, the thermal behavior of 15C5BA and its complexes is studied through polarized optical microscopy (POM) and differential scanning calorimetry (DSC). To understand the effect of the different anions (I^−^, SCN^−^, BF4^−^ and PF6^−^) on the morphology of the ultimate 15C5BA with the different sodium salts, wide‐ and medium angle X‐ray spectroscopy (WAXS and MAXS) is performed.

## Results and Discussion

### ^1^H NMR

**Figure 5:**
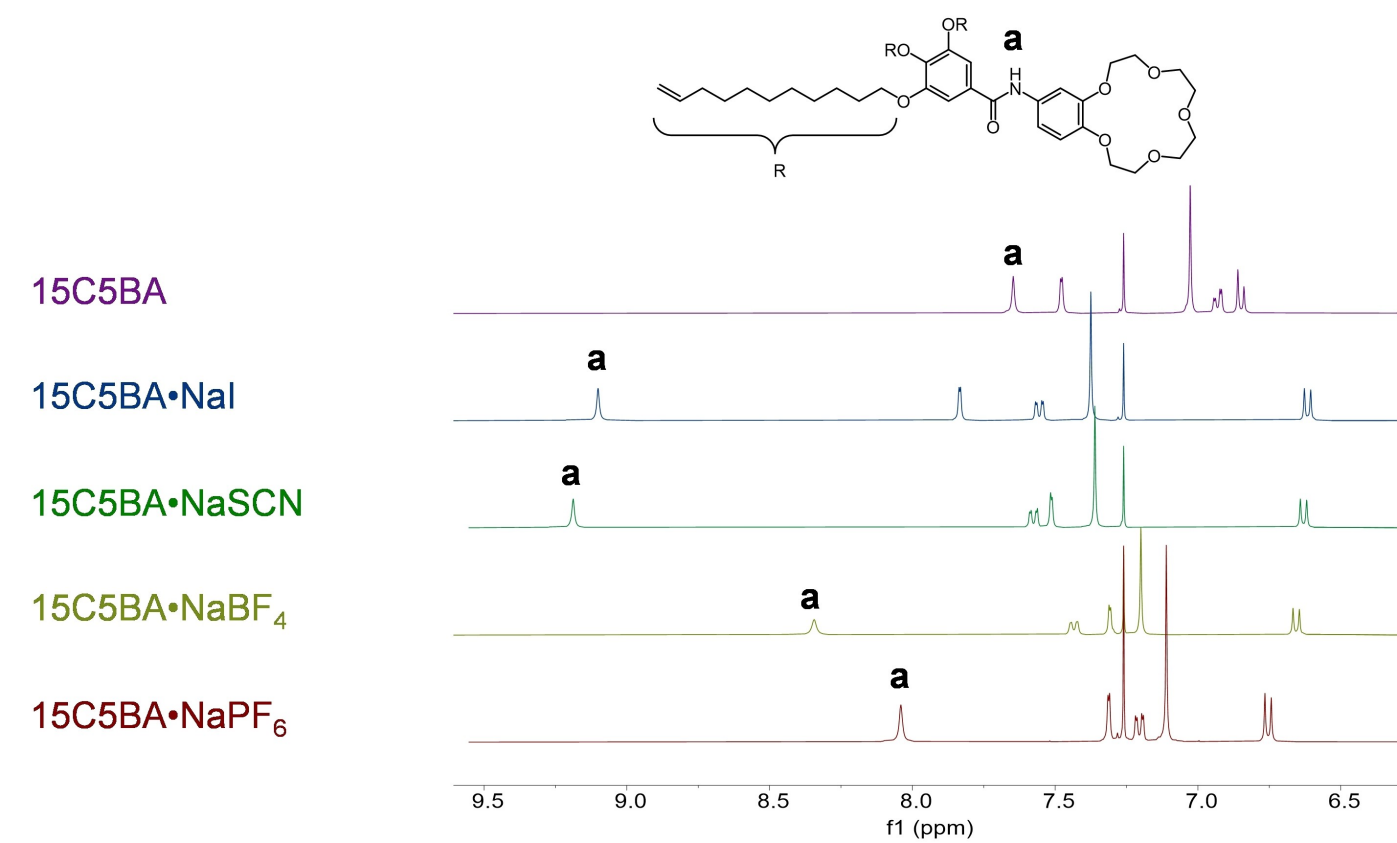
^1^H NMR spectra comparison of 15C5BA and its complexes with the different sodium salts.

^1^H NMR analysis in solution was used to prove the successful synthesis of 15C5BA and its complexation with the different sodium salts (Figure 5). For 15C5BA the signal diagnostic for the presence of an amidic proton is located at 7.65 ppm (proton **a** in Figure 5).

**Figure 6:**
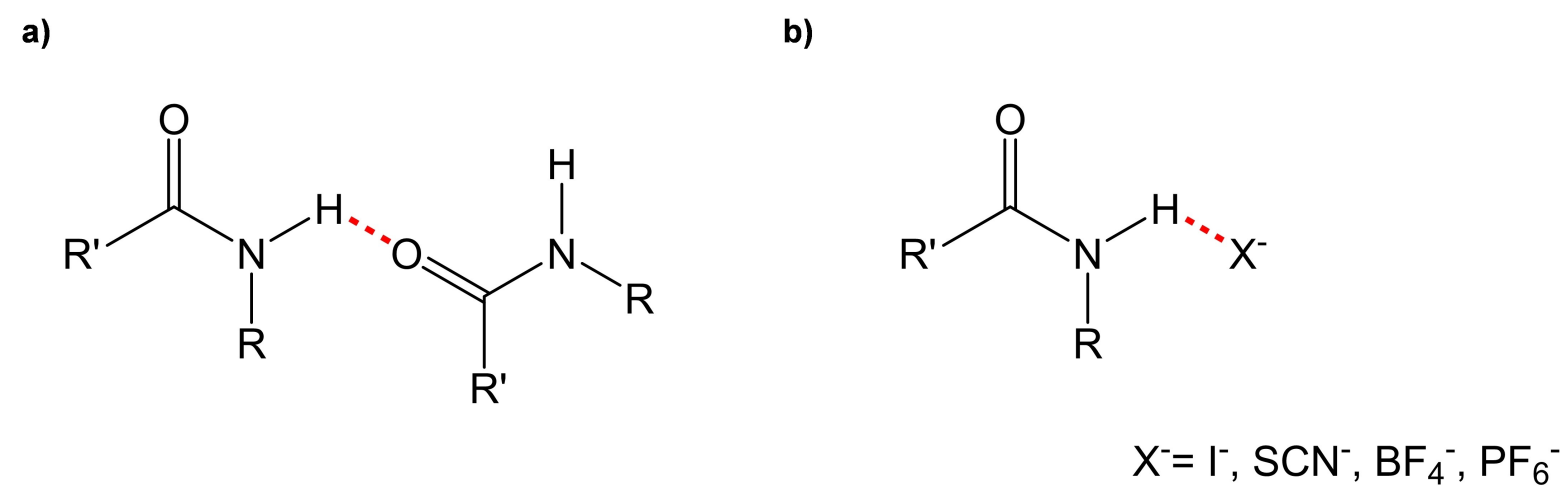
**a)** example of H‐bond between two amidic functionalities, **b)** example of H‐bond between one amidic functionality and an anion.

After complexation with the different sodium salts, significant shifts in this amidic proton signal are observed, indicating that new non‐covalent interactions, such as hydrogen bonds (H‐bonds) and electrostatic interactions, are formed between the anions of the sodium salts (I^−^, SCN^−^, BF_4_^−^ or PF_6_^−^) and the amide itself.[^29^, ^30^, ^31^] Amidic functionalities are known to act as both H‐bond donor (NH group) and acceptor (C=O group).[^32^, ^33^] Based on this, it is hypothesized that before complexation, 15C5BA forms intermolecular H‐bonds between the molecules (Figure 6a) possibly giving rise to a supramolecular assembly.^[34]^

After complexation with the sodium salts, this H‐bond is disrupted by the presence of one of the anions that forms, in turn, a similar interaction with 15C5BA (Figure 6b).^[35]^ The entity of the shift to higher frequencies of the amidic proton of 15C5BA after complexation with the sodium salts can give additional information. This shift can indeed, be attributed to the nature of the anion involved in the interaction with the amide. In particular to the electronegativity, chaotropicity, H‐bond acceptor strength and size of the anion. Table 1 summarizes the findings obtained from the proton NMR regarding the position of the amidic proton in the spectrum.

**Table 1:** Position of amidic proton in ^1^H NMR spectra, electronegativity of anions, anionic radius and chaotropicity.

Entry	Compound	N*H* signal [ppm]	Δδ N*H* [ppm]	Electronegativity anion	Anionic radius [pm]	Chaotropicity ΔHB^[a]^
1	15C5BA	7.65	–	–	–	–
2	15C5BA ⋅ NaI	9.10	1.45	2.66 (I)	206^[36]^	1.37
3	15C5BA ⋅ NaSCN	9.19	1.54	3.04 (N)	213^[37]^	1.03
4	15C5BA ⋅ NaBF_4_	8.35	0.7	2.04 (B)	229^[36]^	1.12
5	15C5BA ⋅ NaPF_6_	8.04	0.39	2.19 (P)	254^[38]^	1.43

[a] number of hydrogen bonds broken in water by the anion.^[36]^

**Figure 7:**
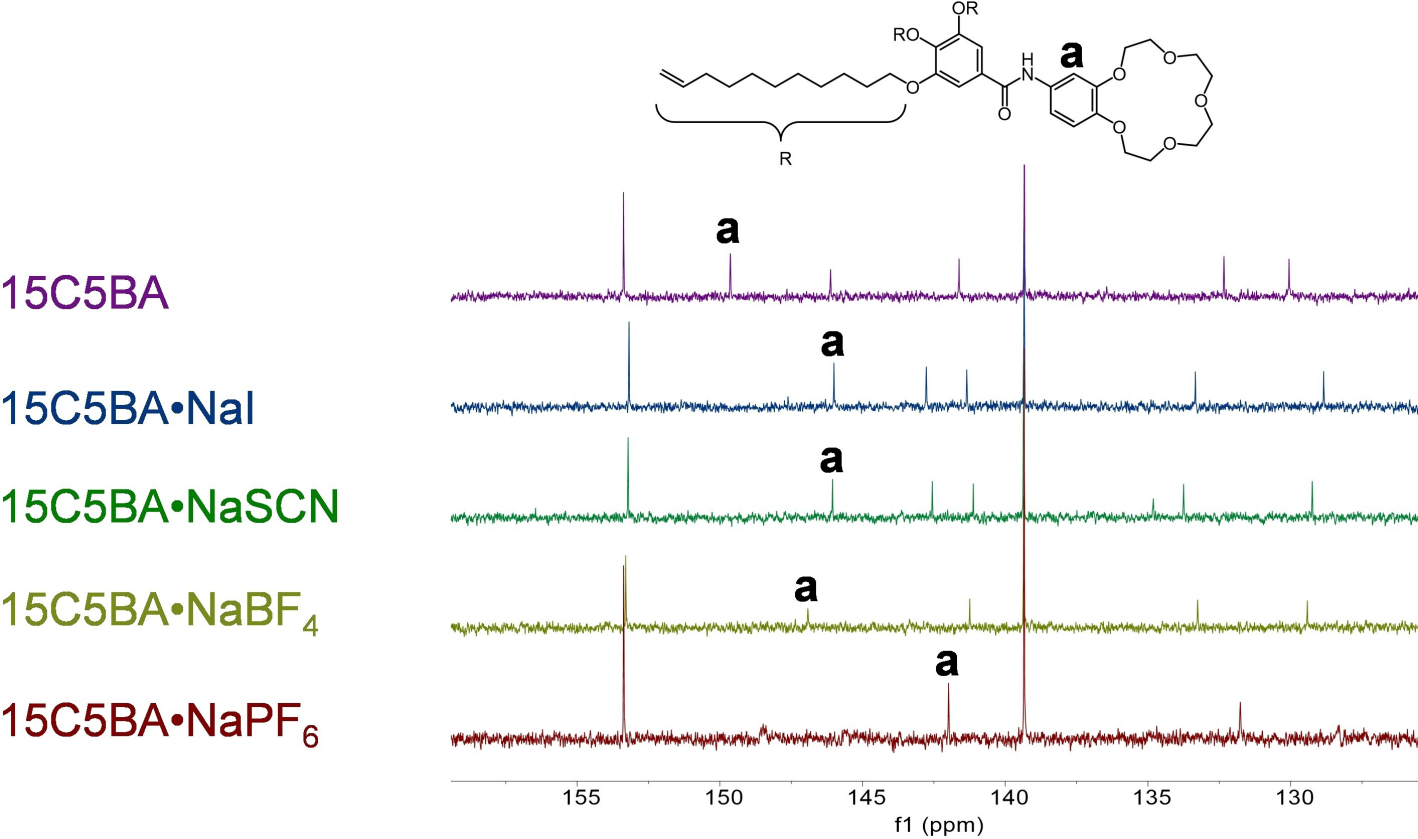
^13^C NMR spectra comparison of the 15C5BA molecule and its complexes with the different sodium salts.

Iodine and nitrogen have the highest electronegativity between the atoms bearing the charge in the anions of the sodium salts. Moreover, those atoms possess free electron pairs making them better H‐bonding acceptors than BF_4_^−^ and PF_6_^−^.[^29^, ^30^] This is clearly confirmed in the significant shift to higher chemical shift (+1.45 ppm for I^−^ and +1.54 ppm for SCN^−^, table 1 entry 2 and 3) observed in their ^1^H NMR spectra for the amidic proton signal. Furthermore, it must be noticed that all the anions used in this study are known for their chaotropicity, hence for their ability in aqueous solutions to break the H‐bonds between water molecules.^[35]^ This effect is known to be responsible for the self‐assembly of some supramolecular systems.[^40^, ^41^, ^42^, ^43^] The data obtained from the ^1^H NMR spectra comparison show an interaction between all the chaotropic anions and the amidic functionality. This supports the hypothesis that the chaotropic ions weaken or even break the intermolecular H‐bonds between 15C5BA pairs of molecules to give rise to similar non‐covalent interactions which involve the presence of the anions themselves (Figure 7b). The deshielding of the signal attributed to the amidic proton does not linearly increase with increasing chaotropicity of the anion. This is explained by taking the size of the anions into account. It is believed that the anions need to access the cavity formed by two 15C5BA molecules and to disrupt the H‐bond between them to be able to interact with the amidic groups. As in the case of PF_6_^−^ and BF_4_^−^, the size of the anion can be a determinant factor for the formation of those H‐bonds. Moreover, PF_6_^−^ and BF_4_^−^ are known to be weekly coordinating anions due to the delocalization of their charge.[^29^, ^39^, ^44^] This can lead to weaker interactions with the amidic functionality. Finally, ^1^H NMR analysis allows an estimation of the spatial proximity of the anions to the amidic region and the higher the deshielding of a signal due to H‐bond, the closer the donor and acceptor involved in H‐bond.^[31]^ This suggests that the closest anion to the amide group is SCN^−^. Considering that the NMR analysis is carried out in the liquid state, the interactions observed may be influenced by the presence of the solvent. Nevertheless, the use of a non H‐bonding solvent (CDCl_3_) for the measurement makes sure that its presence does not interfere with the formation of these intermolecular interactions. These findings prove that, as predicted in the design stage of the non‐globular 15C5BA system, several non‐covalent interactions come into play and their nature and strength are influenced by the complexation with the sodium salts.

### ^13^C NMR

The successful complexation of the sodium salts is also confirmed via ^13^C NMR. Figure 7 shows the spectra of the different complexes. The signal attributed to carbon “a” (as indicated in Figure 7) is found to be at 149.95 ppm and the assignment is confirmed through HSQC experiments (see also SI, Figure S4.1). The signal shifts to lower chemical shifts after complexation with the sodium salts. This shielding effect is clearly due to the presence of the sodium salts. More specifically to the presence of tight ion pairs[^45^, ^46^] between the ions in the salt also due to the non polar nature of the NMR solvent used. Furthermore, similarly to ^1^H NMR the different entity of this shift in the complexes is due to the different H‐bonding ability of the anions of the sodium salts.

This brings to different distances between the anion and the 15C5BA molecule and as a consequence, different magnetic environment around the nuclei and different chemical shift.

### ATR FT‐IR

**Figure 8:**
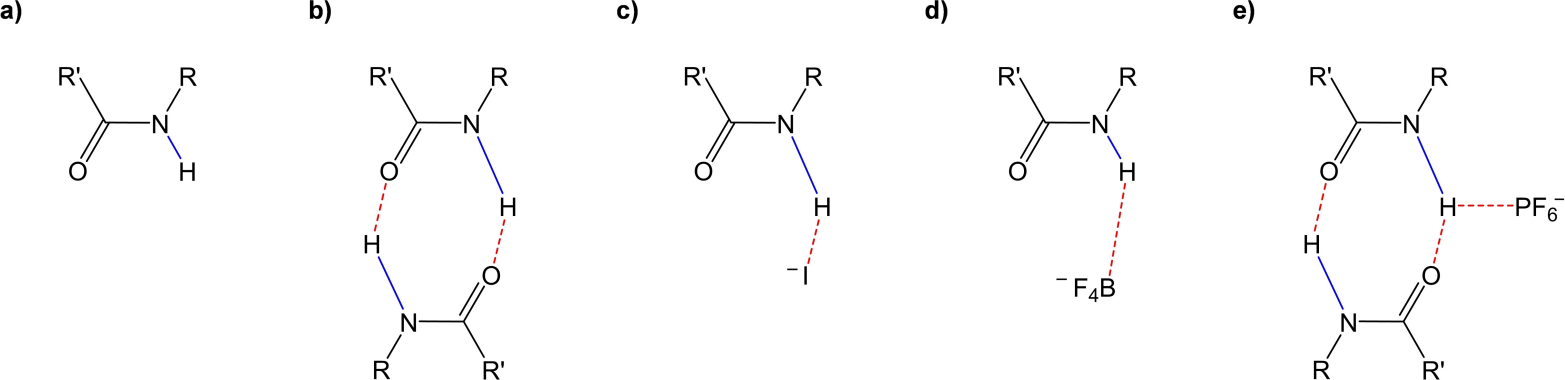
Schematic representation of **a)** free amide **b)** H‐bonds between two amides **c)** H‐bond between amide and I^−^ **d)** H‐bond between amide and BF4^−^ **e)** H‐bond between amide and PF6^−^.

The successful formation of the amidic bond in 15C5BA is confirmed also via FT‐IR measurements. The presence of the signal at 3238 cm^−1^ is attributed to the stretching vibration of the N−H bond while the signal at 1690 cm^−1^ can be associated with the stretching of the carboxylic group (SI, Figure S7.1). Usually, the stretching frequency of the amidic N−H group can be found at higher values.^[47]^ For 15C5BA the low stretching frequency found for the signal attributed to the N−H bond is explained through the formation of intermolecular H‐bonds between 15C5BA molecules. In this situation, the proton is shared between two amidic functionalities (Figure 8b).

**Figure 9:**
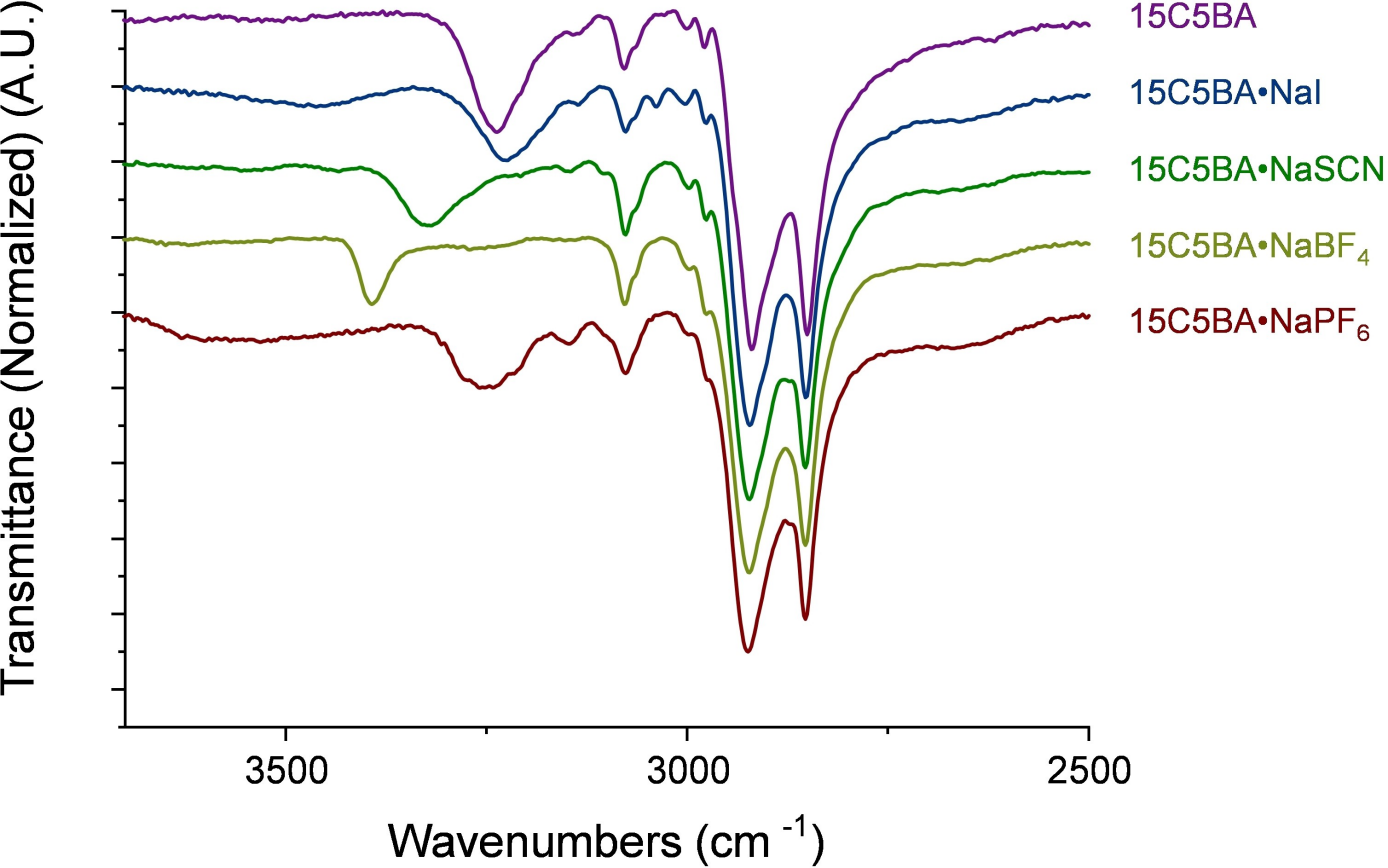
ATR FT‐IR spectra of the 15C5BA molecule and its complexes with the different sodium salts.

As a consequence, the length of the N−H bond (highlighted in blue in Figure 8) is bigger thus, vibrates at lower frequencies. The stretching frequency of the N−H bond of the 15C5BA ⋅ NaI is similar to the one of the uncomplexed 15C5BA (Figure 9).

This translates into a situation in which the amidic proton is shared between 15C5BA and I^−^ with a consequent elongation of the N−H bond (Figure 8c). A shift to higher frequencies of the N−H signal is instead, observed after complexation of 15C5BA with NaSCN and NaBF_4_ (Figure 9). This is due to the disruption of intermolecular H‐bonds between 15C5BA molecules and to the formation of interactions with SCN^−^ or BF_4_^−^ (Figure 8d, complex with NaBF_4_ represented as example). Those interactions are not as strong as in the case of I^−^ and do not allow for a significant elongation of the N−H bond. As a matter of fact, the entity of the shift of the vibrational frequency results from the proximity of the H atom to the donor site.^[48]^ This means that the bigger the shift, the closer the proton to the H‐bond donor (N atom). In addition to this, the results obtained from the FT‐IR, as in the case of the ^1^H NMR, are explained by the strength of the anions studied in terms of their H‐bond acceptor capability.[^29^, ^39^] Further confirmation of the interaction between the amide group and the anions results from the FT‐IR spectrum of 15C5BA ⋅ NaSCN complex (Figure S7.2). The band at 2029 cm^−1^ is attributed to the C=N stretching in the thiocyanate molecule. This band shifts to lower frequencies thus indicating an elongation of the C=N bond. This serves as further confirmation that the anion is involved in an H‐bond with the amide group of 15C5BA. The behaviour shown in the IR for 15C5BA ⋅ NaPF_6_ deviates from the previous discussion. The stretching frequency for the N−H bond, hence the strength of the bond, of this complex is indeed similar to the one of 15C5BA (Figure 9). This correlates with the weak H‐bond acceptor nature of the PF_6_^−^ anion.[^29^, ^39^] As confirmed by ^1^H NMR (Figure 5), the anion PF_6_^−^ has the weakest interaction with the amidic proton of 15C5BA due to its non‐coordinating nature. Even though there is still some interaction with PF_6_^−^, this does not seem to be sufficient to break the H‐bonds between the two amidic functionalities, resulting in a similar stretching frequency as the uncomplexed 15C5BA (Figure 8e).

The results obtained are in agreement with the NMR data and prove that all the anions studied (I^−^, SCN^−^, BF_4_^−^ and PF_6_^−^) as chaotropes can weaken or disrupt the strong H‐bond^[36]^ between the amidic functionalities. Those anions give then rise to interactions of different strength with the amide in the 15C5BA molecule based on their H‐bond acceptor abilities. As previously mentioned, the weakening of the strong amide‐amide H‐bonds, which restrict molecular rotation,[^27^, ^28^] and the formation of less strong interactions with the sodium salts can play a central role in the formation of an ordered assembly of the molecules and thus the facilitate OIPC properties regardless of their non‐globular nature.

### Thermal Behaviour

The thermal behaviour of the synthetized compounds is studied through DSC and summarized in Table 2. 15C5BA shows a monotropic transition before melting (Table 2, SI Figure S5.1).

**Table 2:** Transition temperatures, enthalpies and entropies of 15C5BA and its sodium salt complexes.

Entry	Molecule	Phase transition	Temperature [°C]	ΔH [J ⋅ mol^−1^]	ΔS [J ⋅ K^−1^ ⋅ mol^−1^]
1	15C5BA	S^a^→Iso^b^	79.54; 88.89^c^	43.07	–
2	15C5BA ⋅ NaI	III→II	1.73	2.71	9.85
3		II→I	41.01	3.90	12.41
4		I→Iso	191.49	19.02	40.93^d^
5	15C5BA ⋅ NaSCN	II→I	−24.68	6.85	27.57
6		I→Iso	140.36	24.28	58.73^d^
7	15C5BA ⋅ NaBF_4_	III→II	−20.07	9.02	35.65
8		II→I	42.09	1.99	6.30
9		I→Iso	134.19	23.82	58.48^d^
10	15C5BA ⋅ NaPF_6_	–	–	–	–

a) Solid phase with no or unclear OIPC behaviour. b) Isotropic state. c) This transition involves two, partially resolved transitions between 79 °C and 89 °C. The peak temperatures and the total enthalpy are listed. d) Entropy of fusion.

The transition is observed during the heating cycle and coincides with the appearance of fan‐shaped domains visualized with the POM (SI, Figure S6.1). The formation of these domains indicates that the 15C5BA molecule assembles in columnar arrangements. This is supported by the structural characteristic of 15C5BA which contains an amide bond and two aromatic groups (Figure 3). The first is responsible for the formation of hydrogen bonds and the latter gives rise to π‐π stacking.^[49]^ Together they allow the formation of intermolecular interactions permitting the formation of columnar self‐assembled structures as observed with POM. These results do not support the formation of an OIPC phase for the non‐globular 15C5BA molecule opposite to the other complexes of 15C5BA with sodium salt studied. As previously mentioned, OIPC systems are characterized by the appearance of one or more solid‐solid phase transitions before melting. The absence of such transitions in DSC unambiguously demonstrates that no degrees of freedom are activated before melting, hence no disorder is achieved.

In our case, as proven by NMR and FT‐IR, the absence of such increased degree of disorder before melting for the native 15C5BA molecule is simply due to the formation of strong H‐bonds between the amidic functionalities of the 15C5BA molecules thus preventing molecular rotation. The strength of these interactions is confirmed by the high enthalpy of the transition for the 15C5BA molecule (43 J ⋅ mol^−1^) compared to the ones of the other systems studied in literature (ΔH in Table 2). The non‐globular nature of the native 15C5BA molecule together with the strong H‐bonds thus prevent molecular rotation and the achievement of orientational freedom. Opposite to the native 15C5BA molecule, the complexes of 15C5BA with the different sodium salts show a different thermal behaviour. For the complex of 15C5BA and NaPF_6_ the DSC analysis did not show any phase transitions (SI, Figure S5.5). Nevertheless, the POM of 15C5BA ⋅ NaPF_6_ shows the appearance of small fan‐shaped domains (SI, Figure 6.5). This could be due to a phase transition that involves a small enthalpic contribution due to similar order between phases (molten and phase I). In the case of the molecule with NaI, NaSCN and NaBF_4_ instead, the occurrence of multiple phase transitions is observed before melting (Table 2, see also SI). All these complexes appear as non‐flowing waxes before melting thus disfavouring the hypothesis of a more liquid crystalline nature of the systems.

**Figure 10:**
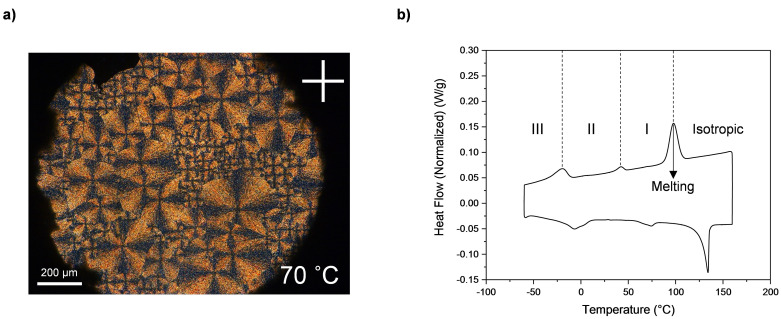
**a)** POM picture of 15C5BA ⋅ NaBF_4_ at 70 °C showing birefringence and the formation of fan shapes. The magnification is 5× and the polarizers are oriented 90° to each other. **b)** DSC thermogram of 15C5BA ⋅ NaBF_4_. The three solid‐solid phase transitions (III, II and I) are clearly indicated.

The solid‐solid transitions visible are accompanied by the formation of fan‐shaped textures showed using POM, indicating the columnar self‐assembly of the molecules[^50^, ^51^] (Figure 10a, complex with NaBF_4_ reported as an example). The formation of plastic phases in columnar systems (“plastic columnar”) has been previously reported in literature.[^52^, ^53^, ^54^] In these cases the stacking of the molecules gives indeed rise to the formation of the columns and consequently, the local rotation of the molecules around the columnar axis allows the formation of the plastic columnar phase. In those systems the formation of spherulitic textures visible ate the POM indicates the organization into columns.

The solid‐solid phase transitions (III, II and I, Figure 10b) introduce an increasing amount of disorder with increasing temperature. Moreover, the complexes with the aforementioned sodium salts show interesting behaviour upon melting. It is indeed noticed that the calculated entropy of fusion for the complexes with NaI, NaSCN and NaBF_4_ (Table 2) is relatively low compared to that of crystalline solids.^[55]^ This finding indicates that before melting the complexes already possess some disorder. This arises from the solid‐solid phase transitions and it suggests a plastic crystal behaviour.^[9]^ Confirmation of the higher plasticity of these systems compared to that of the native 15C5BA molecule is given by the disruption of the strong H‐bonds between the amides and to the formation of weaker bonds with the anions shown with NMR and FT‐IR. This is necessary for the non‐globular system to achieve orientational freedom by overcoming the energy barrier bound to its shape. Nevertheless, unlike already studied plastic crystalline systems, the compounds prepared in this work do not adhere to the Timmermans criterion for which the entropy of fusion of plastic crystals should be below 20 J ⋅ K^−1^ ⋅ mol^−1^.[^9^, ^19^]

The lowest entropy of fusion found is the one for the complex with NaI and it amounts to 40 J ⋅ K^−1^ ⋅ mol^−1^ (Table 2), while in all the other cases the entropy of fusion is higher than indicated by Timmermans. This can be attributed to the complex nature of the complexes studied in this work compared to the relatively simple structures of already known plastic crystals. Similarly, higher entropy values were earlier found for other OIPCs as well[^9^, ^18^] when just one of the two components of the salt exhibits rotator motions, while the other component possesses internal degrees of freedom that are destroyed only after melting.^[17]^ For 15C5BA complexes, it is hypothesized that like in the work of MacFarlane et al., the molecules possess additional degrees of freedom which can be activated upon melting.^[18]^ This can be the case for organic ionic plastic crystals (OIPCs) that are non‐globular and/or formed by two different molecular ions in which just one of the two can experience rotator motions. The complexes prepared in this work consist indeed of two molecular ions. The first one being the anion of the different sodium salts (I^−^, SCN^−^, BF_4_^−^ and PF_6_^−^) and the second one formed by the host‐guest interaction between the non‐globular 15C5BA molecule and Na^+^. The higher entropy of fusion of such systems can also be explained by the formation of residual intermolecular interactions between the 15C5BA complexes. Regarding this matter, it is known that the hydrogen bonds between amide groups are strong in nature and are, for example, responsible for the high melting points of polyamides. This brings us to the conclusion that the energy requirements to break the intermolecular hydrogen bonds between the 15C5BA molecules can also be the determining factor that makes the entropy of fusion higher than as expected by Timmermans for plastic crystalline systems.

### MAXS and WAXS

To obtain further confirmation of the OIPC character of the compounds prepared and to have a better understanding of the effect of the different sodium salts on the order and orientation of such systems, WAXS and MAXS analysis at different temperatures were performed. The large number of sharp scattering signals visible in the diffraction patterns of all the compounds (SI, section 8), typical for an OIPC character,^[10]^ confirms the higher degree of order of the 15C5BA family compared to liquid crystals. Moreover, the presence of more diffuse scattering (SI, section 8) compared to crystalline solids confirms the soft nature of the compounds studied in our work. It is relevant to notice that, all the complexes prepared show minimal to no changes in the scattering patterns obtained at different temperatures (SI, section 8). This suggests that no lattice modification takes place during the phase transitions (Table 2) thereby supporting the hypothesis of positional order. These findings, together with the evidence of the weakening intermolecular interactions after complexation (as shown with NMR and FT‐IR) and the appearance of two or more solid‐solid phase transitions (Table 2), strongly suggest the formation of OIPC phases.

**Figure 11:**
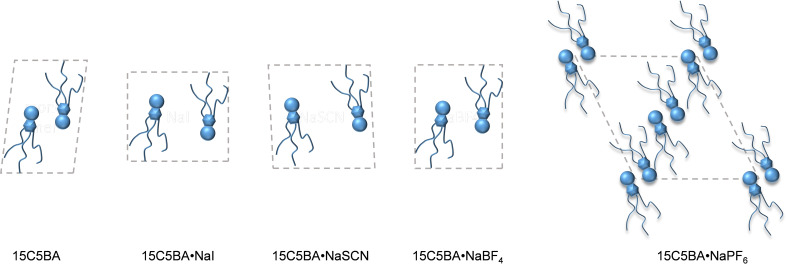
Pictographic representation of the unit cell of 15C5BA and its complexes with different sodium salts.

Interestingly, the complexation with the different sodium salts gives rise to significant differences in terms of morphology. The results obtained are summarized in Table 3 below. From the lattice parameters found, is evident that 15C5BA has the smallest unit cell of all the compounds studied. This is justified by the formation of dimers of 15C5BA molecules (Figure 11 and Figure 8b) due to the H‐bond between their amidic functionalities and confirmed by the FT‐IR results. The complex with NaI, NaSCN and NaBF_4_ result in a bigger unit cell (Table 3).

**Table 3:** Anionic radii and lattice parameter of 15C5BA and its complexes.

Entry	Compound	Anionic radius [Å]	MW [g/mol]	Unit cell	a [Å]	b [Å]	c [Å]	γ [°]	V_cell_ [*10^−24^ cm^3^]
1	15C5BA	–	892.27	Oblique	42.46	28.21	3.60	78	4.24
2	15C5BA ⋅ NaI	2.06	1042.16	Rectangular	33.74	38.23	3.59	90	4.63
3	15C5BA ⋅ NaSCN	2.13	973.34	Oblique (almost square)	38.53	39.2	3.68	103	5.39
4	15C5BA ⋅ NaBF_4_	2.29	1060.22	Rectangular	39.17	33.01	3.81	90	4.92
5	15C5BA ⋅ NaPF_6_	2.54	1002.06	Hexagonal	51.38	51.38	3.71	120	8.48

This is attributed to the disruption of the H‐bond between 15C5BA molecules and the formation of the interactions with the corresponding anions of the sodium salts (Figure 11, see also Figure 8c and 8d). The morphology of the complex with NaSCN is almost square (Table 3, Figure 11).

Since the two axis for this system are very similar and the angle within the unit cell is close to 90°, the difference between the refractive indices is expected to be low. This is confirmed by the absence of birefringence visible in the temperature range of the mesophase at the POM (SI). A significant difference in morphology is noticed for the complex 15C5BA ⋅ NaPF_6_. As previously mentioned, it is assumed that due to the anion size (Table 3) and its non‐coordinating nature, PF_6_^−^ cannot interfere with the strong hydrogen bond between two amidic functionalities (Figure 8e). Therefore, this anion is not able to intercalate between two 15C5BA molecules and it remains as a filler in the crystal system. This behaviour is also shown at FT‐IR in which no effect on the stretching frequency of the NH bond is shown after complexation with NaPF_6_. Moreover, further confirmation is obtained by the size of the unit cell found via WAXS and MAXS analysis. The system 15C5BA ⋅ NaPF_6_ has, indeed, the biggest unit cell between the studied complexes.

## Conclusions

The results obtained in this study verify that globularity is not a necessary condition for the obtainment of an OIPC phase. Even though the use of non‐globular molecules disfavours the arising of orientational freedom due to the lack or symmetry and hindered rotation, the typical plasticity of OIPCs can still be obtained by finely tuning the intermolecular interactions. The strength of those interactions plays a key role in the achievement of orientational freedom. If the intermolecular interactions are too strong, such as the amidic H‐bonds in the native 15C5BA molecule in our work, the formation of the rotator phase is prevented. This is aligned with previously reported literature[^1^, ^28^] and translates into the formation of a system that can have both positional and orientational order with no plasticity. On the other hand, we demonstrated that the strength of such interactions can be tuned and the presence of weaker interactions is crucial to give rise to the characteristic orientational freedom of the OIPC phase while still allowing the formation of positional order. Successful weakening of the strong H‐bond interactions can be achieved through complexation of the 15C5BA molecule with different sodium salts containing chaotropic anions. The formation of the non‐covalent interactions between 15C5BA and the sodium salts and the weakening of the H‐bonds between the amide functionalities in our work, is indeed confirmed by ^1^H NMR and FT‐IR measurements. The complexation is driven by the presence of the 15‐crown‐5 ether moiety in the molecular structure. The non‐covalent interactions between Na^+^ and the ether linkages form the foundation on which the OIPC system is built. This allows for the systematic study of the effect of different chaotropic anions on the properties of an OIPC system. We clearly observe through DSC measurements, that the OIPC phase is obtained when using anions that can effectively break the H‐bond between the amides (I^−^, SCN^−^ and BF_4_^−^). The interaction with these anions is proven to be effective in the achievement of orientational freedom despite the non‐globularity of the 15C5BA system. Moreover, the complexes studied show different morphologies when analysed through MAXS and WAXS. By simply tuning the nature of the sodium salts we successfully obtained a new non‐globular OIPC family and are able to control the thermal and morphological properties of these systems. This approach will help to significantly widen the scope of OIPC materials to more complex systems designed to tailor specific functionalities for emerging applications.

## Experimental Section

**Scheme 1:**
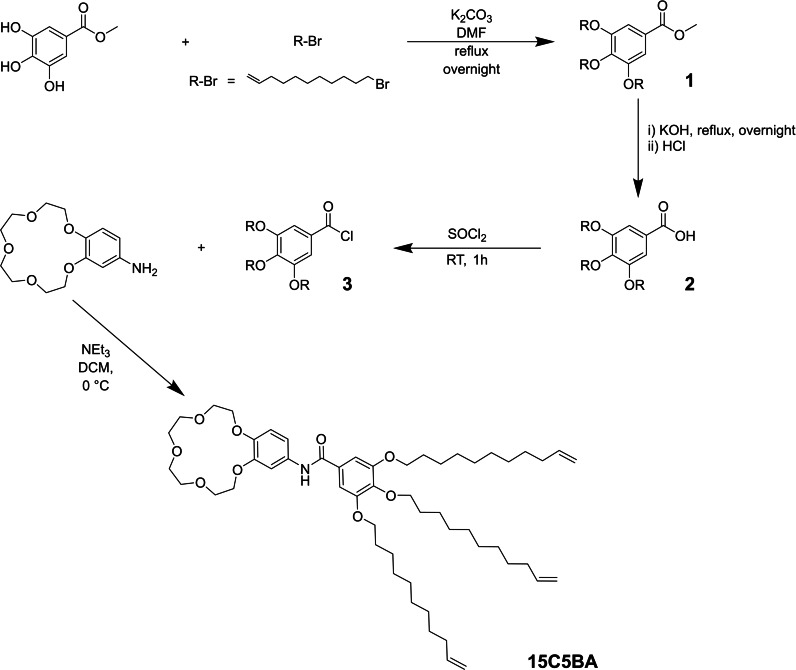
reaction scheme for the synthesis of 15C5BA.

The following syntheses were performed (Scheme 1):

**Synthesis of Compound 1**: After three cycles vacuum/nitrogen, 1 g of methyl 3,4,5‐trihydroxybenzoate (5.43 mmol, 1 eq) was dissolved in dry DMF. Thereafter, 7.51 g of K_2_CO_3_ (54.30 mmol, 10 eq) was added and the slurry was stirred at RT for 10 minutes. After this time, 3.93 mL of 11‐bromo‐1‐undecene (17.92 mmol, 3.3 eq) was added and the obtained mixture was refluxed overnight at 120 °C under an inert atmosphere. While increasing the temperature of the reaction mixture, a colour change of the solution from transparent to light brown was observed. The reaction was stopped after 12 h by cooling down the reaction mixture to room temperature. The excess K_2_CO_3_ was removed via gravimetric filtration and the filtrate was evaporated to dryness through rotary evaporation yielding a brown dense liquid. The obtained product was dissolved in EtOAc (40 mL) and extracted 3 times with water (40 mL×3). The organic phase was collected, dried over Na_2_SO_4_ and filtrated gravimetrically. The filtrate was evaporated to dryness to obtain a light brown oil. The crude product was purified with flash chromatography (Heptane:EtOAc, elution gradient from 1 : 0 to 4 : 1) obtaining pure compound 1 as a white wax with a yield of 2.78 g (80 %). ^1^H NMR (400 MHz, CDCl_3_, 25 °C, TMS): δ (ppm)=1.30–1.51 (m, 36H), 1.70–1.85 (m, 6H), 2.0–2.07 (m, 6H), 3.89 (s, 3H, O‐C*H*_3_), 4.91–5.01 (dd, 6H, *J*=10 Hz and 17 Hz, 3×C*H*_2_=CH), 5.76–5.82 (ddt, 3H, *J*=7 Hz, 10 Hz and 17 Hz, 3×CH_2_=C*H*‐CH_2_), 7.25 (s, 2H, *H*_ar_). ^13^C NMR (100 MHz, CDCl_3_, 25 °C, TMS): δ (ppm)=25.74, 28.95, 28.98, 29.15, 29.20, 29.31, 29.37, 29.42, 29.45, 29.54, 29.57, 29.65, 30.33, 33.93, 52.09, 69.17, 73.46, 108.02, 114.12, 124.66, 139.18, 142.38, 152.81, 166.93.

**Synthesis of Compound 2**: The synthesis of compound **2** was performed following a previously reported procedure.^[56]^ In a 100 mL round bottom flask, 2 g of compound **1** (3.12 mmol, 1 eq) was dissolved in a mixture of H_2_O/MeOH (1 : 3, 40 mL). Subsequently 1.75 g of KOH, 31.20 mmol, 10 eq) was dissolved in the same solvent mixture and added dropwise to the system. The combined mixture was then heated to reflux at 75 °C under magnetic stirring, overnight. The system was then allowed to cool down to room temperature and neutralized to pH≈3 with HCl (3 M). The solution was then mixed with EtOAc (50 mL) and extracted 3 times with brine (50×3 mL). The organic phase was dried with Na_2_SO_4_ and evaporated to dryness via rotary evaporation to obtain pure compound **2** as a white wax with a quantitative yield. ^1^H NMR (400 MHz, CDCl_3_, 25 °C, TMS): δ (ppm)=1.28–1.50 (m, 36H), 1.72 (m, 6H), 2.01–2.06 (m, 6H), 3.85–3.3.99 (m, 6H), 4.91–5.01 (dd, 6H, *J*=10 Hz and 17 Hz, 3×C*H*_2_=CH), 5.75–5.85 (ddt, 3H, *J*=7 Hz, 10 Hz and 17 Hz, 3×CH_2_=C*H*‐CH_2_), 7.21 (s, 2H, *H*_ar_). ^13^C NMR (100 MHz, CDCl_3_, 25 °C, TMS): δ (ppm)=26.13, 26.22, 29.00, 29.23, 29.43, 29.57, 29.67, 29.73, 30.43, 33.84, 68.99, 73.39, 108.19, 114.14, 139.13, 152.67. ATR FT‐IR (Neat, cm^−1^): 3076, 2922, 2851, 1686, 1585, 1429, 1327, 1225, 1119, 991, 907, 770, 721.

**Synthesis of Compound 3**: In a 50 mL round bottom flask with two necks 1.5 g of compound **2** (2.39 mmol, 1 eq) was added. After creating an inert atmosphere with 3 cycles of vacuum/nitrogen, 2.95 mL of SOCl_2_ (40.67 mmol, 17 eq) slowly added with a syringe and the system was stirred for 1 h at room temperature. Subsequently, the excess of SOCl_2_ was removed using a vacuum pump for approximately 2 h, which quantitatively yielded compound **3** as a dark brown oil. Product **3** was used in the following reaction steps without further purification.

**Synthesis of Compound 15C5BA**: First, 0.5 g of 4′‐aminobenzo‐15‐crown‐5 (1.76 mmol, 1 eq) was added in a round bottom flask with two necks. Three cycles vacuum/nitrogen were performed to apply an inert atmosphere. The brown solid was dissolved in approximately 20 mL of dry DCM. The system was kept in an ice bath at 0 °C under magnetic stirring for 5 min. To this solution, 271 μL of NEt_3_ (1.94 mmol, 1.1 eq) was added. After 15 min, a solution of 1.25 g of compound **3** (19.41 mmol, 1.1 eq) in approximately 10 mL of dry DCM was added dropwise to the reaction flask under magnetic stirring. Stirring was continued under an inert atmosphere subsequently at 0 °C for 2 h and at room temperature for 16 h. Completion of the reaction was confirmed with TLC. The crude product was extracted 3 times with H_2_O (3×40 mL). The organic phase was subsequently dried with Na_2_SO_4_ and filtrated via gravimetric filtration. The excess DCM was removed with rotary evaporation. The brown sticky solid obtained was further purified by recrystallization in the minimum amount of warm EtOAc (∼15 mL). This system was cooled down in the fridge overnight (∼16 h) and the solid product obtained was separated via Buchner filtration obtaining compound **15C5BA** as a pale grey solid with a yield of 74 % (1.17 g). ^1^H NMR (400 MHz, CDCl_3_, 25 °C, TMS): δ (ppm)=1.30–1.50 (m, 36H), 1.69–1.86 (m, 6H), 1.98–2.09 (m, 6H), 3.70–3.80 (m, 8H, 2×O‐(C*H*_2crown_)_2_‐O), 3.85–3.93 (m, 4H, 2×O‐C*H*_2crown_‐CH_2crown_‐O−Ar), 3.99–4.04 (m, 6H, 3×O‐C*H*_2_‐CH_2_), 4.09‐4.20 (m, 4H, CH_2crown_‐C*H*_2crown_‐O−Ar), 4.88–5.04 (dd, 6H, *J*=10 Hz and 16 Hz, 3×C*H*_2_=CH), 5.74–5.87 (ddt, 3H, *J*=7 Hz, 10 Hz and 17 Hz, 3×CH_2_=C*H*‐CH_2_), 6.83–6.85 (d, *J*=9 Hz, 1H, O‐C_ar_‐C*H*_ar_‐CH_ar_‐C‐amide) 6.93–6.96 (d, *J*=9 Hz, 2H, O‐C_ar_‐CH_ar_‐C*H*_ar_‐C‐amide), 7.04 (s, 2H, 2×C*H*_ar_ gallate), 7.48 (s, 1H, O−C‐C*H*_ar_‐C‐amide), 7.67 (bs, 1H, NH amide). ^13^C NMR (100 MHz, CDCl_3_, 25 °C, TMS): δ (ppm)=165.92, 153.69, 149.95, 146.44, 141.94, 139.65, 132.66, 130.38, 115.28, 114.59, 112.97, 107.71, 106.23, 74.00, 71.57, 71.49, 71.07, 70.92, 70.16, 69.95, 69.93, 69.33, 34.29, 34.26, 30.77, 30.11, 30.02, 30.00, 29.91, 29.83, 29.82, 29.65, 29.60, 29.43, 29.40, 26.52. ATR FT‐IR (Neat, cm^−1^): 3260, 3076, 2922, 2851, 1751, 1638, 1582, 1516, 1427, 1341, 1231, 1119, 993, 909, 845, 721. (MALDI‐TOF): [M+Na]^+^ calcd. for C_54_H_85_NO_9_: 914.61; found: 914.65.

**General Procedure for the Complexation of Compound 15C5BA with Sodium Salts**: First 50 mg of compound **15C5BA** (0.056 mmol, 1 eq) was dissolved in 2 mL of DCM. Then, 2 equivalents of the chosen sodium salt was dissolved in MeOH. This solution was added to the **15C5BA** solution and a colour change of the solution from brown to red was observed. The complexation was performed overnight at room temperature under magnetic stirring. Subsequently, the mixture was evaporated to dryness via rotary evaporation. The crude solid was redissolved in DCM and the formation of a heterogeneous mixture was observed. This mixture was filtered via gravimetric filtration. The filtrate was collected and evaporated to dryness via rotary evaporation obtaining a sticky solid with quantitative yield.

### 15C5BA ⋅ NaI

^1^H NMR (400 MHz, CDCl_3_, 25 °C, TMS): δ (ppm)=1.30–1.52 (m, 36H), 1.71–1.86 (m, 6H), 2.01–2.06 (m, 6H), 3.69–4.20 (m, 22H, 16×C*H*_crown_ and 3×O‐C*H*_2_), 4.91–5.01 (dd, 6H, *J*=10 Hz and 17 Hz, 3×C*H*_2_=CH), 5.76–5.86 (ddt, 3H, *J*=7 Hz, 10 Hz and 17 Hz, 3×CH_2_=C*H*‐CH_2_), 6.65–6.67 (d, 1H, *J*=9 Hz, O‐C_ar_‐C*H*_ar_‐CH_ar_‐C‐amide), 7.32 (s, 2H, 2×C*H*_ar_ gallate), 7.47–7.49 (dd, *J*=2 Hz and 9 Hz, 1H, O‐C_ar_‐CH_ar_‐C*H*_ar_‐C‐amide), 7.82 (d, *J*=2 Hz, 1H, O−C‐C*H*_ar_‐C‐amide), 8.88 (s, 1H, N*H* amide). ^13^C NMR (100 MHz, CDCl_3_, 25 °C, TMS): δ (ppm)=166.00, 153.19, 146.01, 142.77, 141.35, 139.35, 133.34, 128.85, 114.58, 114.25, 112.38, 107.97, 106.54, 73.61, 69.99, 69.18, 69.06, 68.06, 67.81, 67.18, 33.95, 30.50, 29.82, 29.75, 29.72, 29.69, 29.63, 29.57, 29.34, 29.31, 29.11, 29.09, 26.31, 26.22.

### 15C5BA ⋅ NaSCN

^1^H NMR (400 MHz, CDCl_3_, 25 °C, TMS): δ (ppm)=1.30–1.53 (m, 36H), 1.72–1.88 (m, 6H), 2.01–2.06 (m, 6H), 3.62–4.16 (m, 22H, 16×C*H*_crown_ and 3×O‐C*H*_2_), 4.91–5.02 (dd, 6H, *J*=10 Hz and 17 Hz, 3×C*H*_2_=CH), 5.76–5.87 (ddt, 3H, *J*=7 Hz, 10 Hz and 17 Hz, 3×CH_2_=C*H*‐CH_2_), 6.63–6.65 (d, 1H, *J*=9 Hz, O‐C_ar_‐C*H*_ar_‐CH_ar_‐C‐amide), 7.35 (s, 2H, 2×C*H*_ar_ gallate), 7.52 (s, 1H, O−C‐C*H*_ar_‐C‐amide), 7.55–7.57 (d, *J*=9 Hz, 1H, O‐C_ar_‐CH_ar_‐C*H*_ar_‐C‐amide), 9.16 (s, 1H, N*H* amide). ^13^C NMR (100 MHz, CDCl_3_, 25 °C, TMS): δ (ppm)=165.26, 152.90, 145.74, 142.24, 140.80, 139.04, 139.03, 134.50, 133.44, 128.93, 113.94, 113.53, 112.18, 106.22, 105.76, 73.30, 69.22, 68.85, 67.75, 66.87, 66.73, 33.65, 30.19, 29.51, 29.45, 29.42, 29.38, 29.32, 29.03, 29.01, 28.80, 28.79, 26.00, 25.92.

### 15C5BA ⋅ NaBF_4_

^1^H NMR (400 MHz, CDCl_3_, 25 °C, TMS): δ (ppm)=1.30–1.52 (m, 36H), 1.71–1.86 (m, 6H), 2.01–2.06 (m, 6H), 3.66–4.15 (m, 22H, 16×C*H*_crown_ and 3×O‐C*H*_2_), 4.91–5.01 (dd, 6H, *J*=10 Hz and 17 Hz, 3×C*H*_2_=CH), 5.76–5.86 (ddt, 3H, *J*=7 Hz, 10 Hz and 17 Hz, 3×CH_2_=C*H*‐CH_2_), 6.65–6.67 (d, 1H, *J*=9 Hz, O‐C_ar_‐C*H*_ar_‐CH_ar_‐C‐amide), 7.20 (s, 2H, 2×C*H*_ar_ gallate), 7.32 (s, 1H, O−C‐C*H*_ar_‐C‐amide), 7.43–7.46 (d, *J*=9 Hz, 1H, O‐C_ar_‐CH_ar_‐C*H*_ar_‐C‐amide), 8.32 (s, 1H, N*H* amide). ^13^C NMR (100 MHz, CDCl_3_, 25 °C, TMS): δ (ppm)=165.44, 153.30, 146.92, 141.25, 139.36, 133.26, 129.42, 114.26, 113.22, 112.87, 106.10, 105.70, 77.48, 77.16, 76.84, 73.61, 69.53, 69.41, 68.15, 67.51, 33.96, 30.50, 29.82, 29.75, 29.73, 29.70, 29.63, 29.59, 29.55, 29.35, 29.32, 29.12, 29.10, 26.25.

### 15C5BA ⋅ NaPF_6_

^1^H NMR (400 MHz, CDCl_3_, 25 °C, TMS): δ (ppm)=1.30–1.49 (m, 36H), 1.71–1.84 (m, 6H), 2.01–2.06 (m, 6H), 3.67–4.13 (m, 22H, 16×C*H*_crown_ and 3×O‐C*H*_2_), 4.91–5.01 (dd, 6H, *J*=10 Hz and 17 Hz, 3×C*H*_2_=CH), 5.76–5.86 (ddt, 3H, *J*=7 Hz, 10 Hz and 17 Hz, 3×CH_2_=C*H*‐CH_2_), 6.75–6.78 (d, 1H, *J*=9 Hz, O‐C_ar_‐C*H*_ar_‐CH_ar_‐C‐amide), 7.10 (s, 2H, 2×C*H*_ar_ gallate), 7.16–7.19 (dd, *J*=2 Hz and 9 Hz, 1H, O‐C_ar_‐CH_ar_‐C*H*_ar_‐C‐amide), 7.35 (d, *J*=2 Hz, 1H, O−C‐C*H*_ar_‐C‐amide), 7.98 (s, 1H, N*H* amide). ^13^C NMR (100 MHz, CDCl_3_, 25 °C, TMS): δ (ppm)=166.75, 153.37, 141.99, 139.34, 131.77, 114.26, 107.84, 106.16, 77.48, 77.16, 76.84, 73.71, 70.34, 69.80, 69.53, 69.11, 68.11, 33.97, 33.95, 30.47, 29.81, 29.72, 29.70, 29.61, 29.54, 29.49, 29.34, 29.30, 29.12, 29.09, 26.20.

## Supporting Information

Materials and methods, ^1^H NMR spectra, ^13^C NMR spectra, HSQC spectra, ATR FT‐IR spectrum, DSC thermograms, POM images and MAXS/WAXS data are available free of charge.

## Author Contributions

The manuscript was written through contributions of all authors. All authors have given approval to the final version of the manuscript.

## Notes

The authors declare no competing financial interest.

## Conflict of interest

The authors declare no conflict of interest.

1

## Supporting information

As a service to our authors and readers, this journal provides supporting information supplied by the authors. Such materials are peer reviewed and may be re‐organized for online delivery, but are not copy‐edited or typeset. Technical support issues arising from supporting information (other than missing files) should be addressed to the authors.

Supporting Information

## Data Availability

The data that support the findings of this study are available from the corresponding author upon reasonable request.
