# Supplementary material for: Non‐Globular Organic Ionic Plastic Crystal Containing a Crown‐Ether Moiety – Tuning Its Behaviour Using Sodium Salts
Source: Chemphyschem. 2022 Jun 28;23(15):e202200258. doi: 10.1002/cphc.202200258 (PMC9400962; doi:10.1002/cphc.202200258)
Supplement: Supplementary file 1 — Supporting Information [file CPHC-23-0-s001.pdf]

ChemPhysChem

Supporting Information

Non-Globular Organic Ionic Plastic Crystal Containing a Crown-Ether Moiety – Tuning Its Behaviour Using Sodium Salts

Anna Casimiro, Jody Lugger, Johan Lub, and Kitty Nijmeijer*

Supporting information

Contents

1. Materials and methods 3
2. ^{1}H NMR 5
   2.1. ^{1}H NMR compound 1 5
   2.2. ^{1}H NMR compound 2 5
   2.3. ^{1}H NMR compound 15C5BA 6
   2.4. ^{1}H NMR compound 15C5BA•NaI 6
   2.5. ^{1}H NMR compound 15C5BA•NaSCN 7
   2.6. 1H NMR compound 15C5BA•NaBF4 7
   2.7. ^{1}H NMR compound 15C5BA•$NaPF_6$ 8
3. ^{13}C NMR 8
   3.1. ^{13}C NMR compound 15C5BA 8
   3.2. ^{13}C NMR compound 15C5BA•NaI 9
   3.3. ^{13}C NMR compound 15C5BA•NaSCN 9
   3.4. ^{13}C NMR compound 15C5BA•$NaBF_4$ 10
   3.5. ^{13}C NMR compound 15C5BA•$NaPF_6$ 10
4. HSQC 11
   4.1. HSQC compound 15C5BA 11
   4.2. HSQC compound 15C5BA•NaI 11
   4.3. HSQC compound 15C5BA•NaSCN 12
   4.4. HSQC compound 15C5BA•$NaBF_4$ 12
5. Differential Scanning Calorimetry 13
   5.1. DSC thermogram of 15C5BA 13
   5.2. DSC thermogram of 15C5BA•NaI 13
   5.3. DSC thermogram of 15C5BA•NaSCN 14
   5.4. DSC thermogram of 15C5BA•$NaBF_4$ 14
   5.5. DSC thermogram of 15C5BA•$NaPF_6$ 15
6. Polarized optical microscopy 15
   6.1. POM of 15C5BA at different temperatures (5x magnification) 15
   6.2. POM of 15C5BA•NaI at different temperatures (5x magnification) 15
   6.4. POM of 15C5BA•$NaBF_4$ at different temperatures (5x magnification) 16
   6.5. POM of 15C5BA•$NaPF_6$ at 93 °C (5x magnification) 17
7. ATR FT-IR 17
   7.1. ATR FT-IR of 15C5BA 17
   7.2. ATR FT-IT of 15C5BA•NaSCN 18

8. Medium- and wide- angle X-ray analysis 18
8.1. 1D and 2D diffractograms at different temperatures diffractograms and d values found and calculated for 15C5BA..18
8.4. 1D and 2D diffractograms at different temperatures diffractograms and d values found and calculated for 15C5BA•$NaBF_4$ 24
8.5. 1D and 2D diffractograms at different temperatures and d values found and calculated for 15C5BA•$NaPF_6$..26
9. References: 27

1. Materials and methods

Nuclear magnetic resonance spectroscopy (NMR) spectra were recorded at room temperature on a Bruker, FT-NMR spectrometer AVANCE III HD-Nanobay (400 MHz, Bruker Ultrashield magnet, BBFO Probehead, BOSS1 shim assembly) in deuterated chloroform. Chemical shifts are given in ppm with respect to tetramethyl silane (TMS, 0 ppm) as internal standard.

Matrix-assisted laser desorption/ionization time-of-flight mass spectrometry (MALDI-TOF MS) was performed on a Bruker Autoflex Speed MALDI-MS instrument using DCTB (2-[(2E)-3-(4-tert-butylphenyl)-2-methylprop-2-enylidene] malononitril) as matrix.

Attenuated total reflection Fourier transform infrared spectroscopy (ATR FT-IR) spectra were recorded at room temperature on a Varian-cary 3100 FT-IR spectrometer equipped with a golden gate attenuated total reflectance (ATR) sampling accessory. Scans were taken over a range of 4000−650 cm^{-1}, with a spectral resolution of 4 cm^{-1} and 50 scans per spectrum.

Differential scanning calorimetry (DSC) measurements were recorded in hermetic T-zero aluminium sample pans using a TA Instruments Q2000 DSC equipped with cooling accessory. The DSC measurements were performed, using approximately 5 mg of compound, with three cycles of heating and cooling at a rate of 2 °C/min with an isothermal equilibration of 3 minutes after each heating or cooling ramp. The transition temperatures were determined from the third heating and cooling cycle using TRIOS DSC software.

Polarising optical microscopy (POM) was performed using a Leica DM 2700M optical microscope equipped with two cross polarizers with the sample in between, a Linkam hot-stage THMS600 with a Linkam TMS94 controller and a Leica DFC420 C camera. The samples were heated up till melting and cooled down at 2 °C/min.

Diffraction (XRD) images were recorded on a Ganesha lab instrument equipped with a Genix-Cu ultra-low divergence source producing X-ray photons with a wavelength of 1.54 Å and a flux of 1 x 108 photons/second. Diffraction patterns were collected on a Pilatus 300K detector with reversed-biased silicon diode array sensor. The detector contains 487 x 619 pixels of 172 x 172 μm2 and consists out of three modules with an intermodule gap of 17 pixels in between, resulting in two dark bands on the image. Temperature-dependent measurements were executed with a Linkam HFSX350 heating stage and cool unit. Measurements were performed on bulk samples sealed in 1.0 mm diameter glass capillaries, 0.01-mm-wall thickness (Hilgenberg). Azimuthal integration of the obtained diffraction patterns was performed by utilizing the SAXSGUI software. The beam centre and the q-range was calibrated by utilizing silver behenate (0.107 Å-1; 58.43 Å). D-spacings were calculated from the following

relation: $d = 2\pi / q$. The assignment of the Miller indices has been done manually for the principal peaks, starting from the simplest unit cell, from where the corresponding lattice parameters and interplanar spacings were calculated. This was done in line the plane spacing equations[1] including a monoclinic unit cell, until a suitable unit cell was found, one that accounts for all the observed diffraction peaks. The final lattice parameters were obtained by using a least squares fitting method, as outlined by Herk et al.[2], taking the lattice parameters as variable; where via a non-iterative approach the optimal lattice parameters were determined – fitting the found corresponding d-spacings with the simplest crystal structure. The lattice volume was calculated from the resultant lattice parameters, and the density was calculated by adjusting the amount of molecules contained in the unit cell to have a representative density as a resultant.

Unless specified otherwise, all reagents were purchased by SigmaAldrich, Alfa Aesar, or TCI Europe and used without further purification. All solvents were obtained from Biosolve.

2. ^{1}H NMR

2.1. ^{1}H NMR compound 1

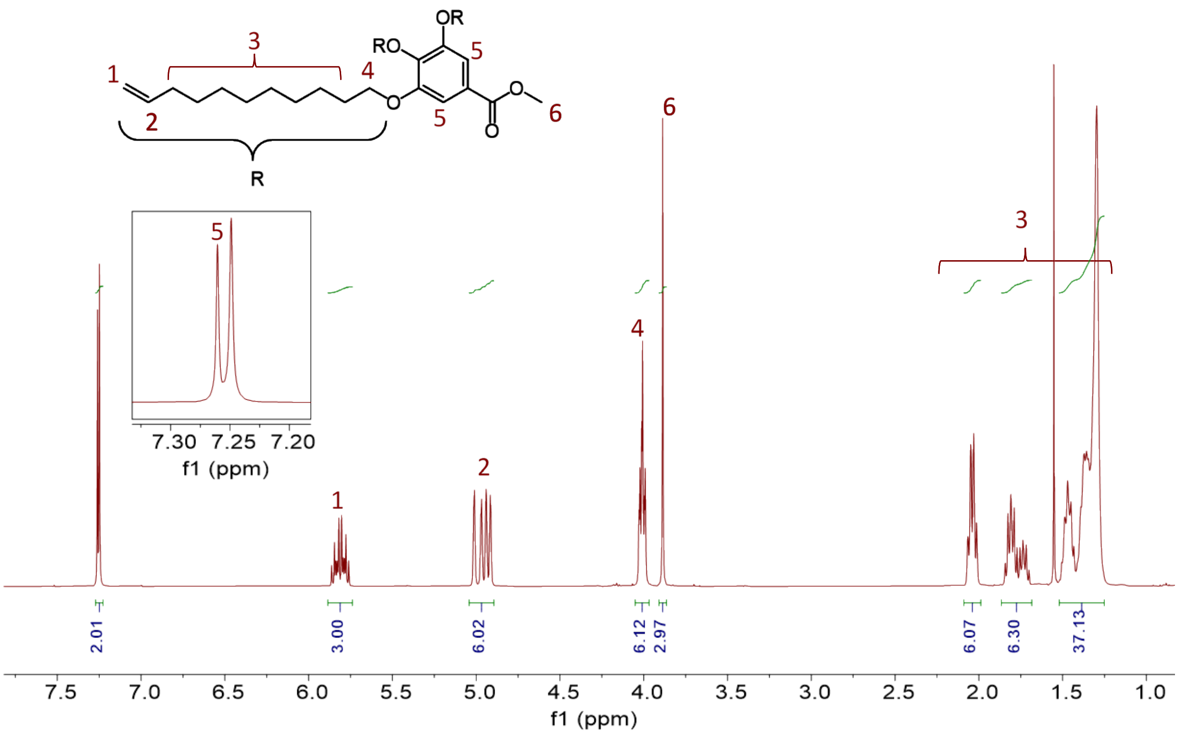

2.2. ^{1}H NMR compound 2

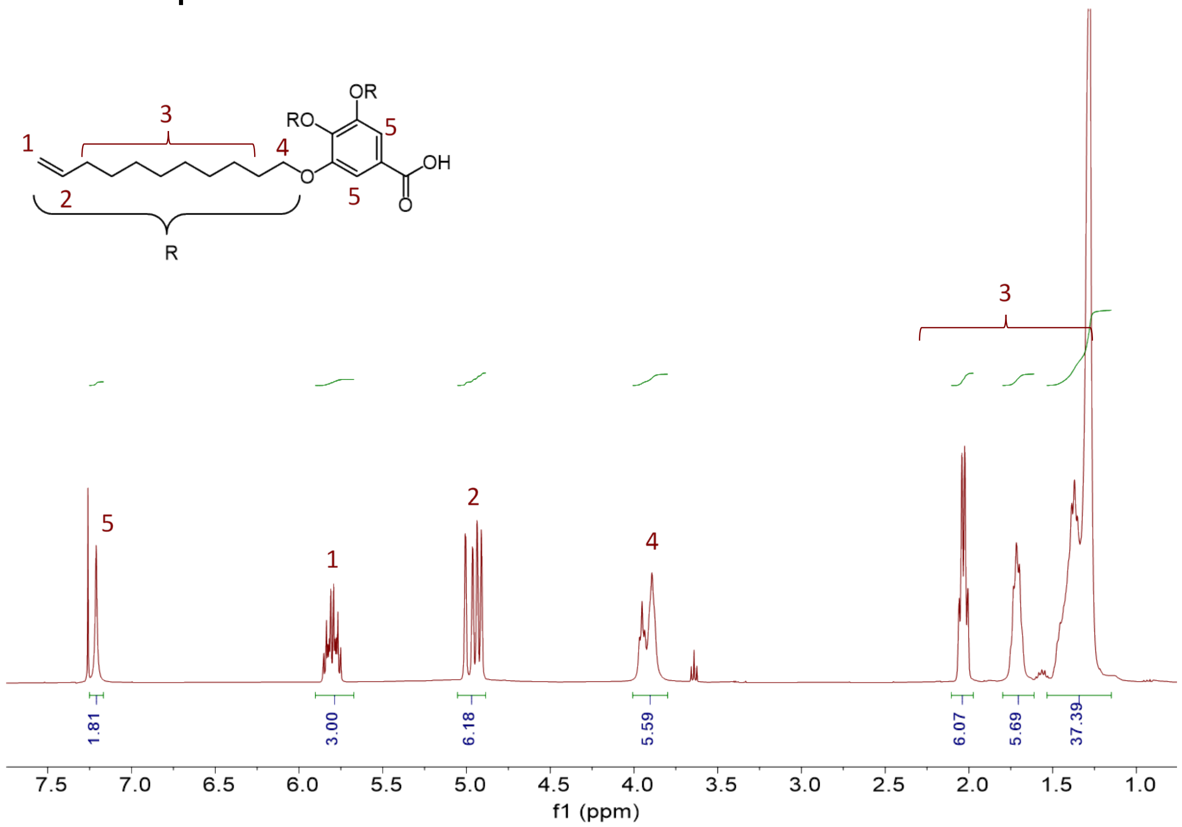

2.3. ^{1}H NMR compound 15C5BA

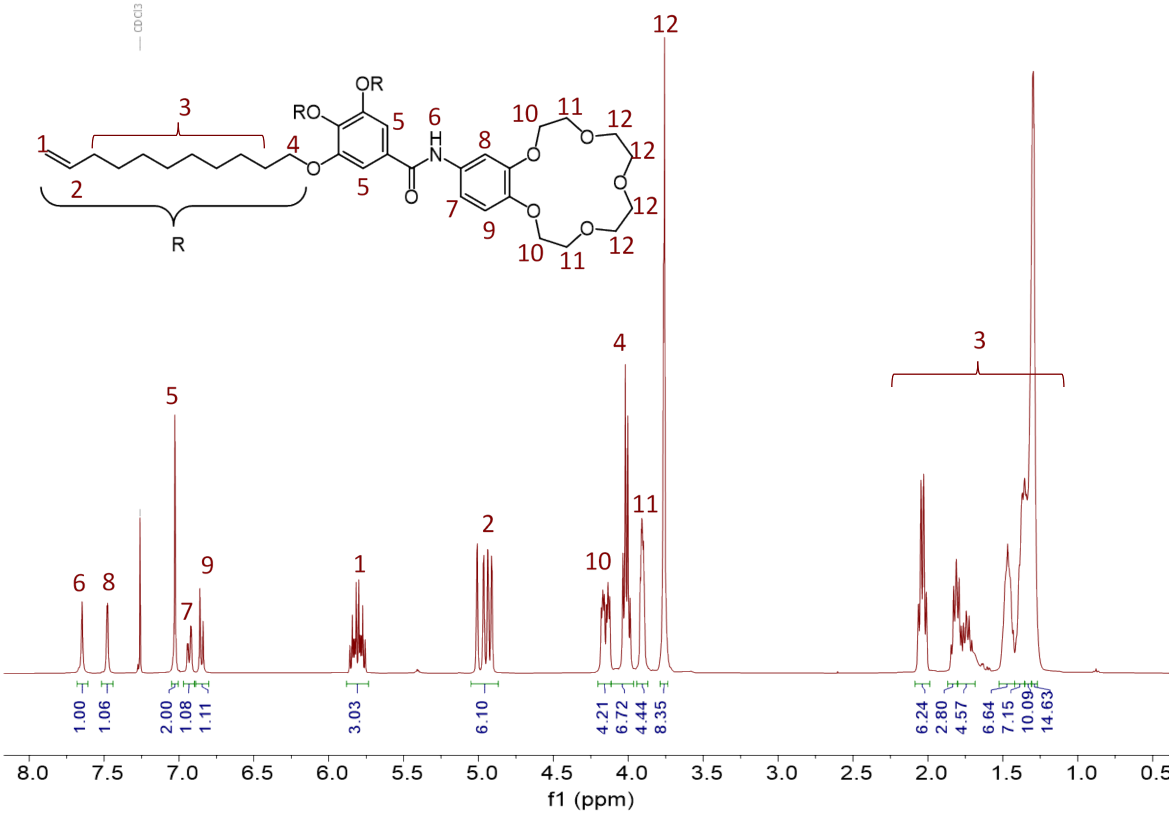

2.4. ^{1}H NMR compound 15C5BA•NaI

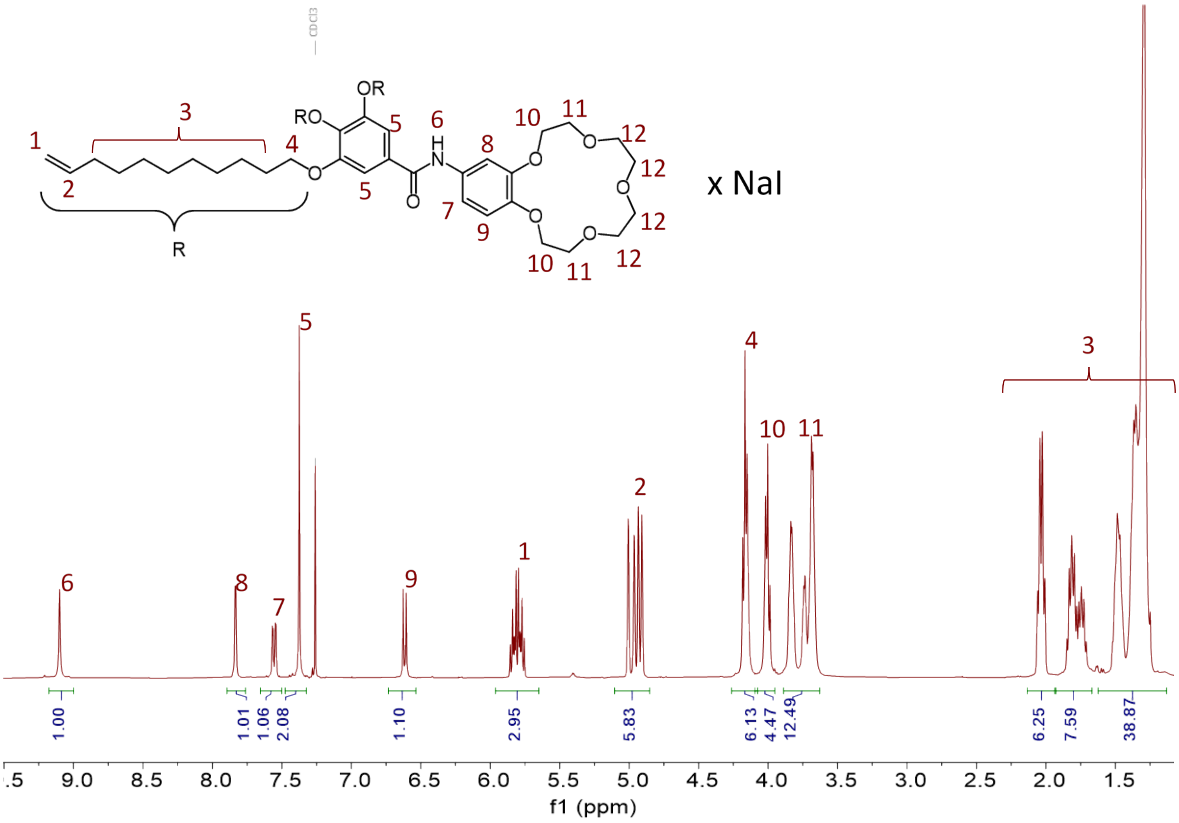

2.5. ^{1}H NMR compound 15C5BA•NaSCN

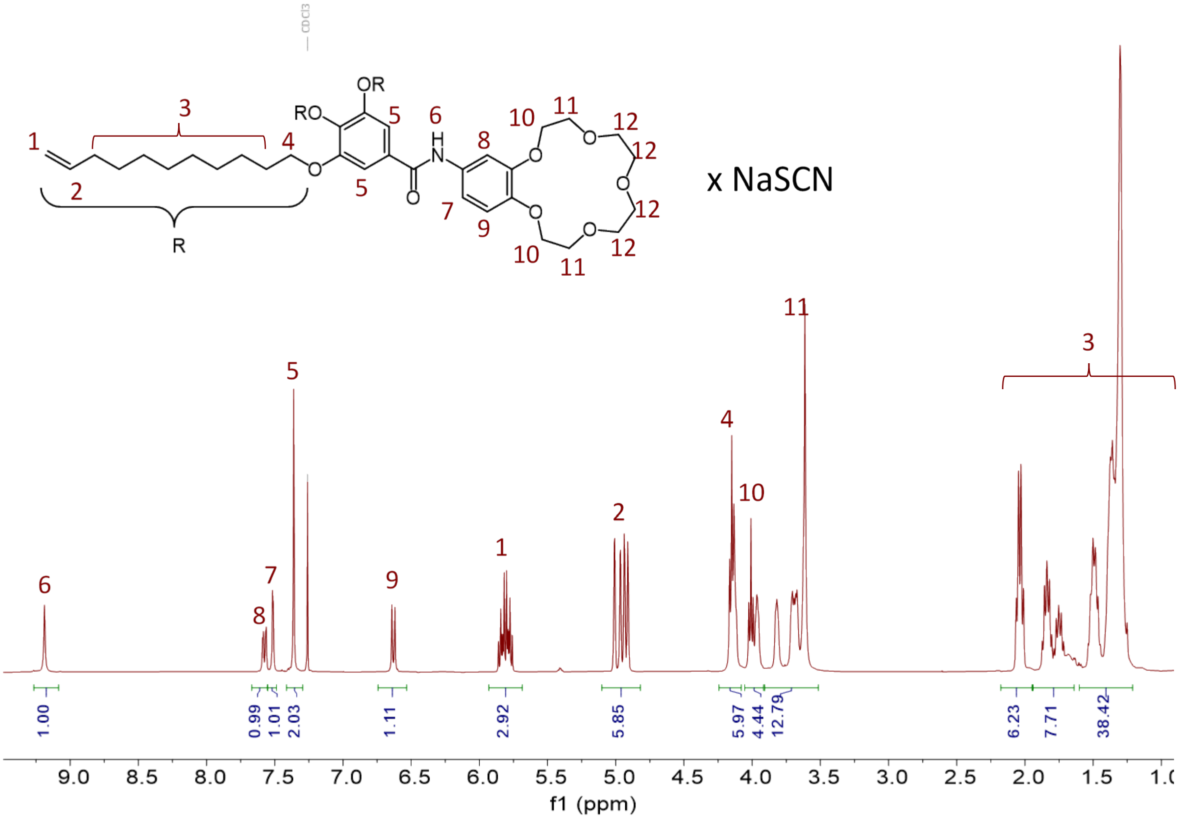

2.6. 1H NMR compound 15C5BA•NaBF4

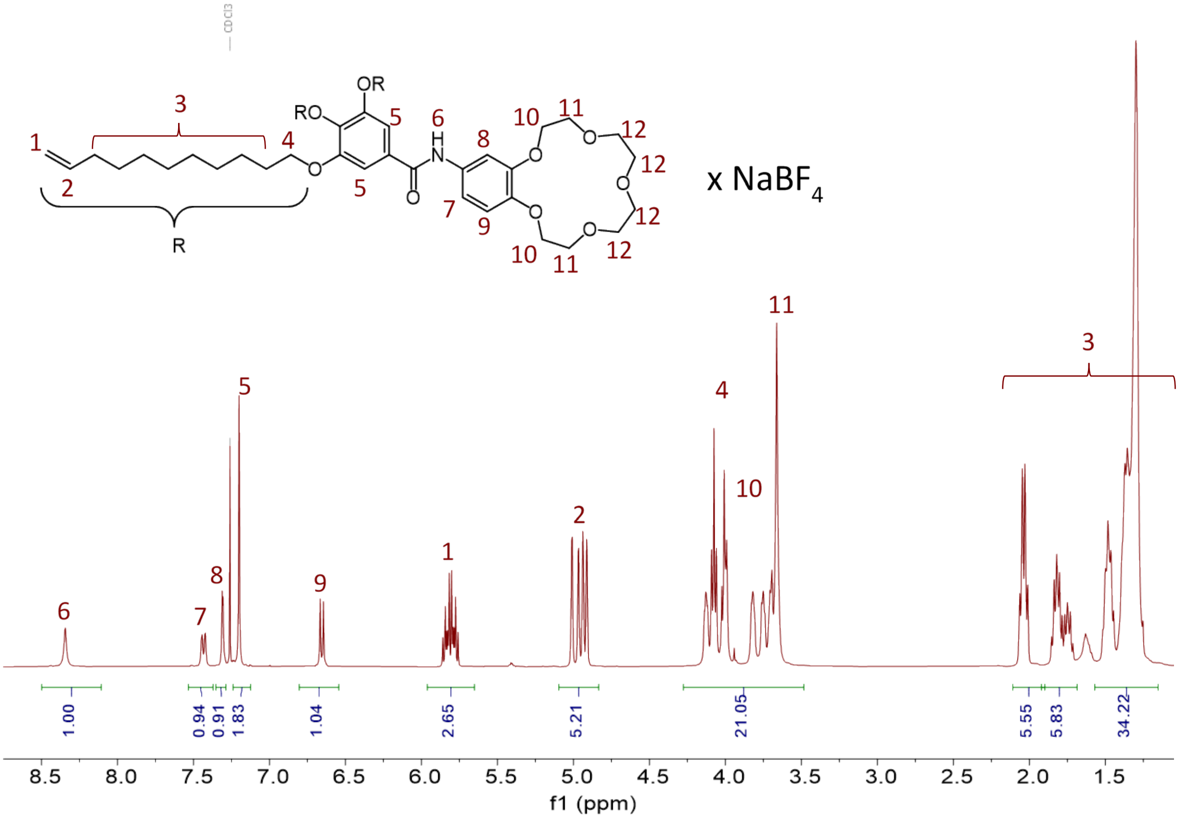

2.7. ^{1}H NMR compound 15C5BA•$NaPF_6$

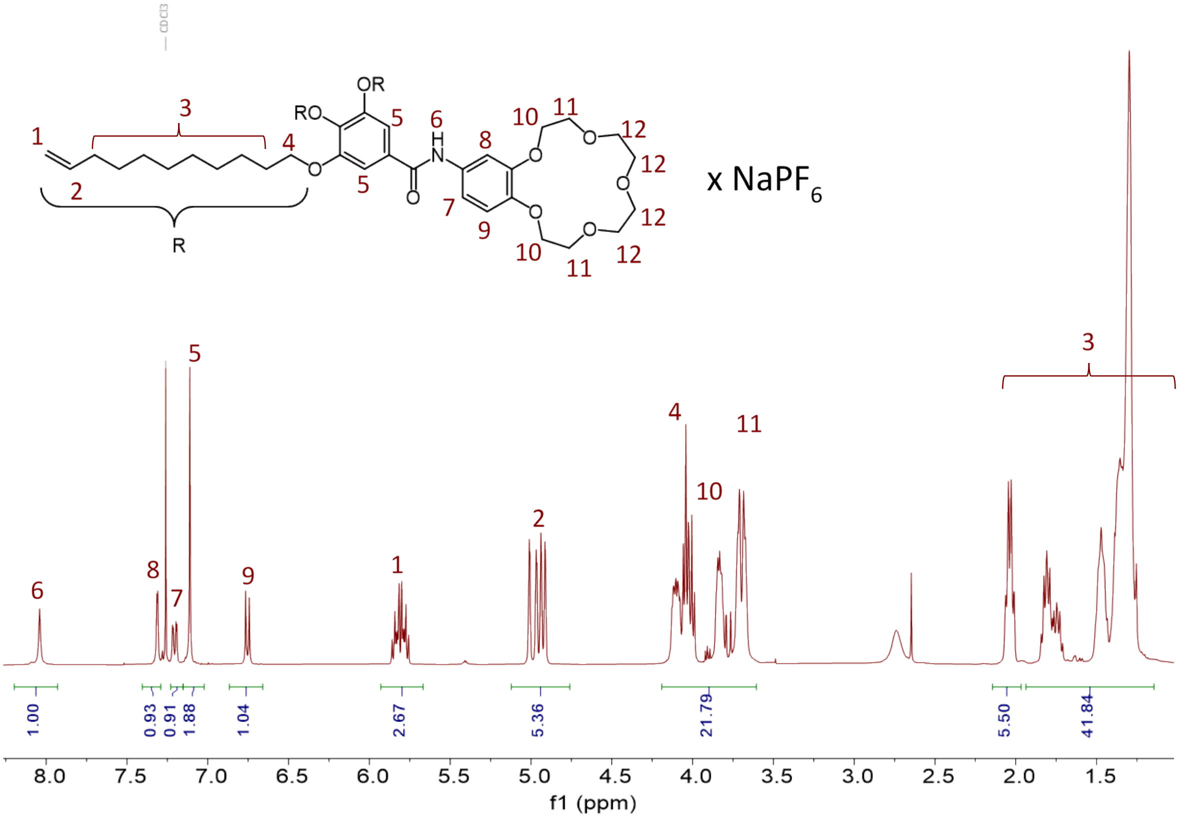

3. ^{13}C NMR

3.1. ^{13}C NMR compound 15C5BA

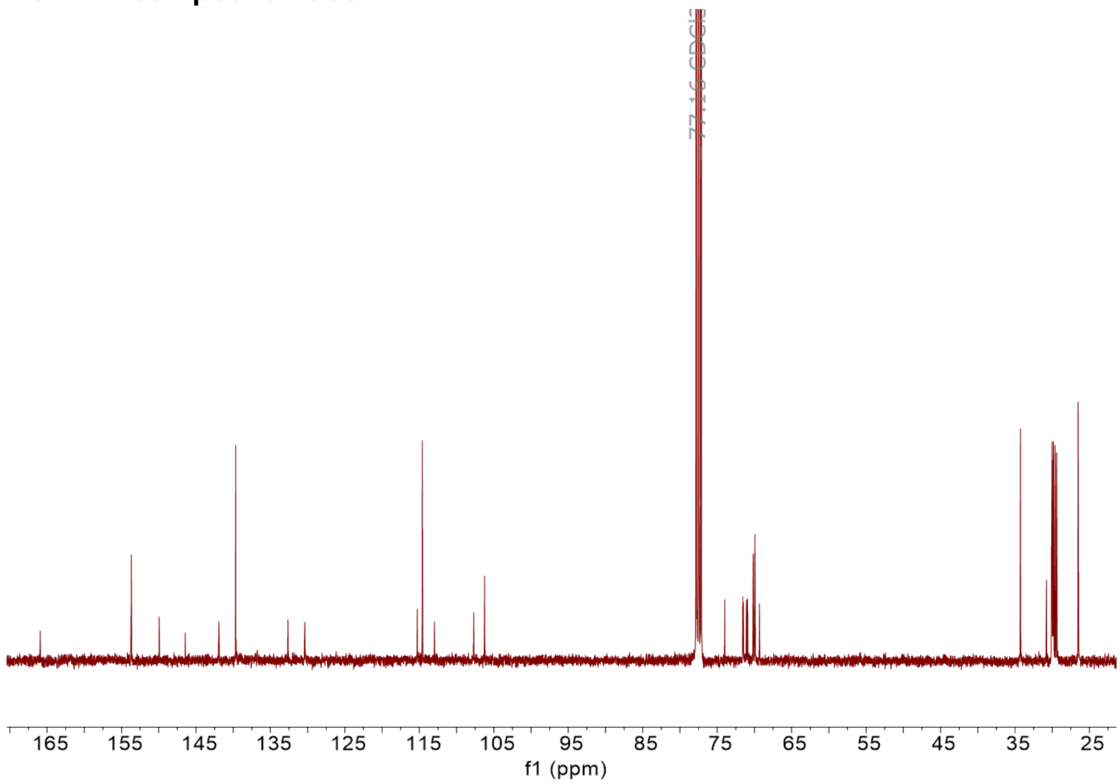

3.2. ^{13}C NMR compound 15C5BA•NaI

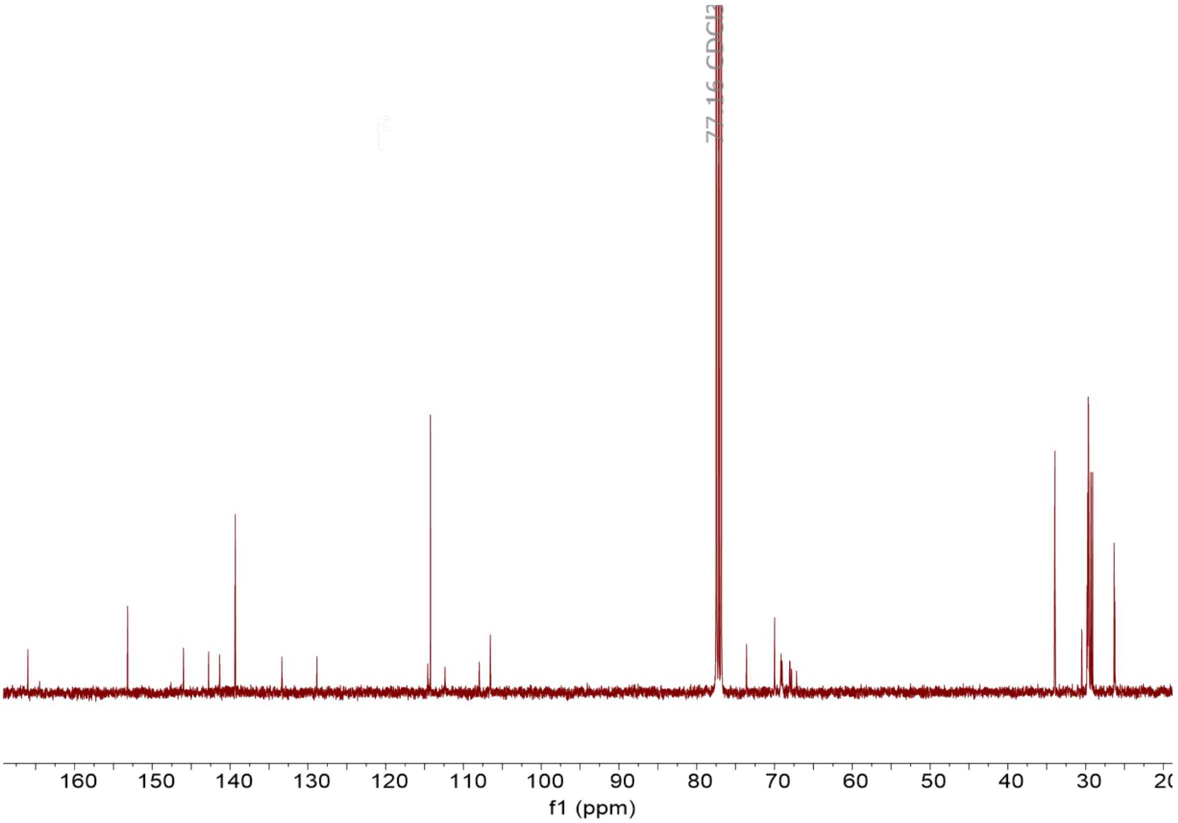

3.3. ^{13}C NMR compound 15C5BA•NaSCN

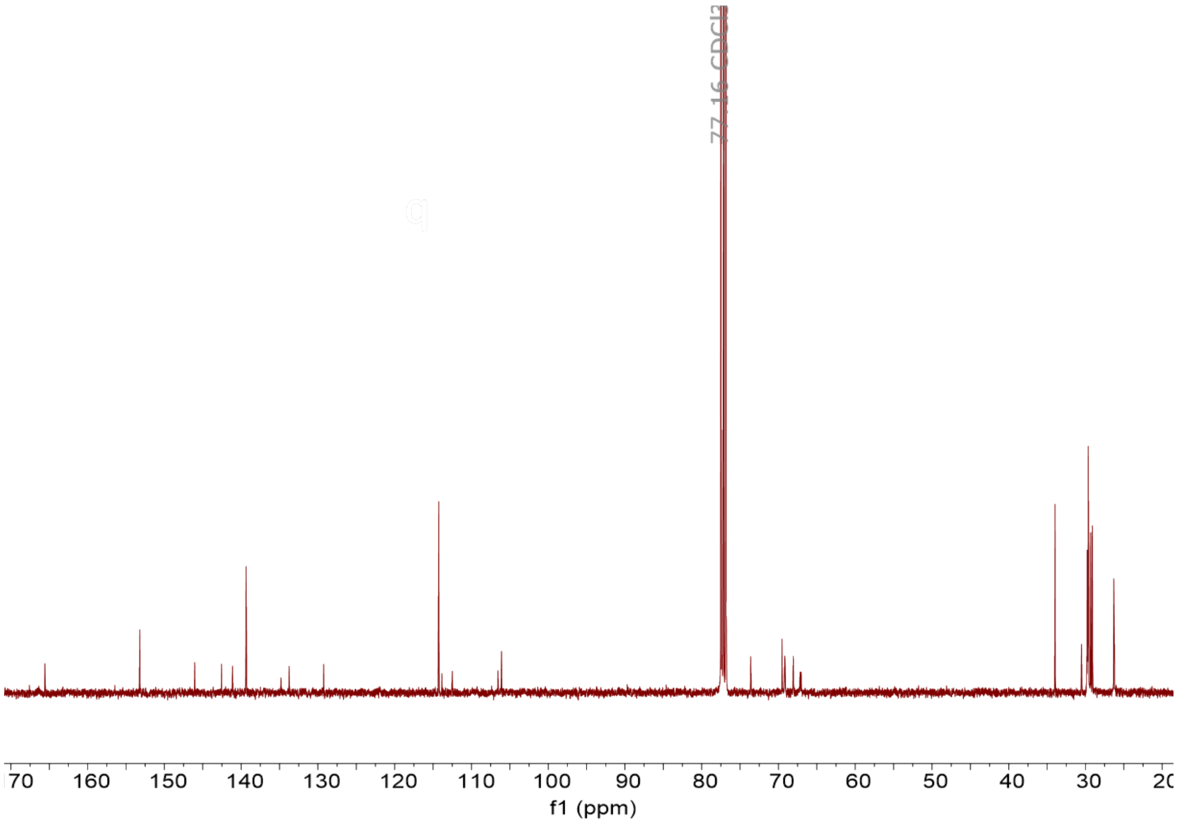

3.4. ^{13}C NMR compound 15C5BA•$NaBF_4$

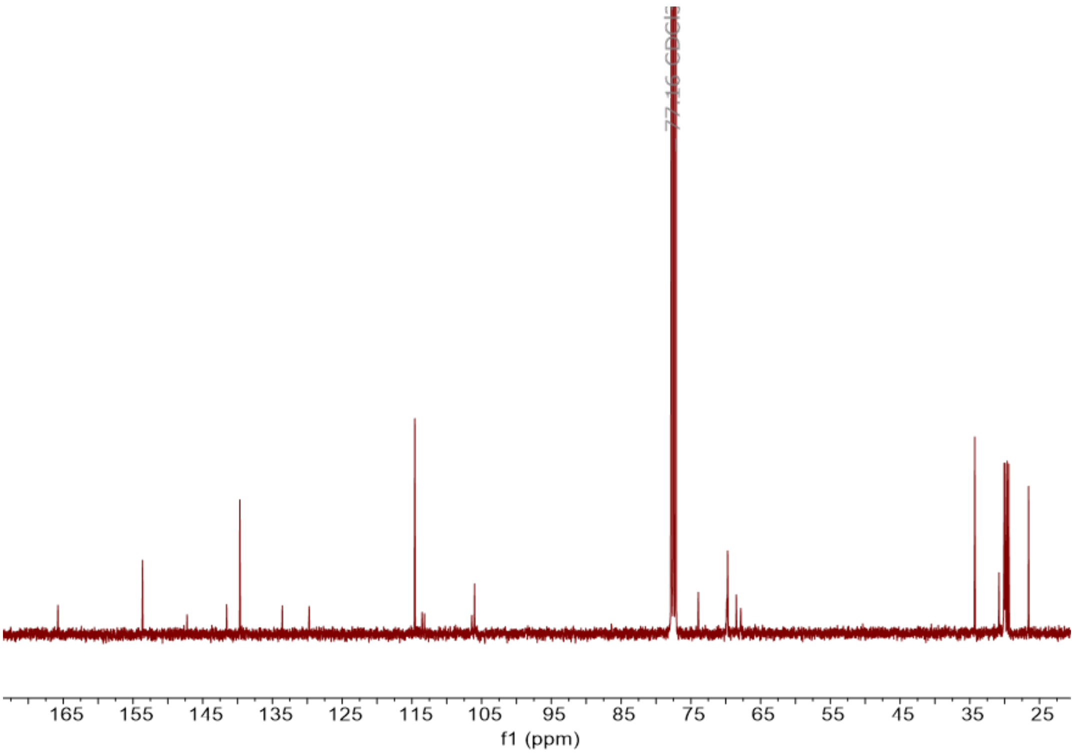

3.5. ^{13}C NMR compound 15C5BA•$NaPF_6$

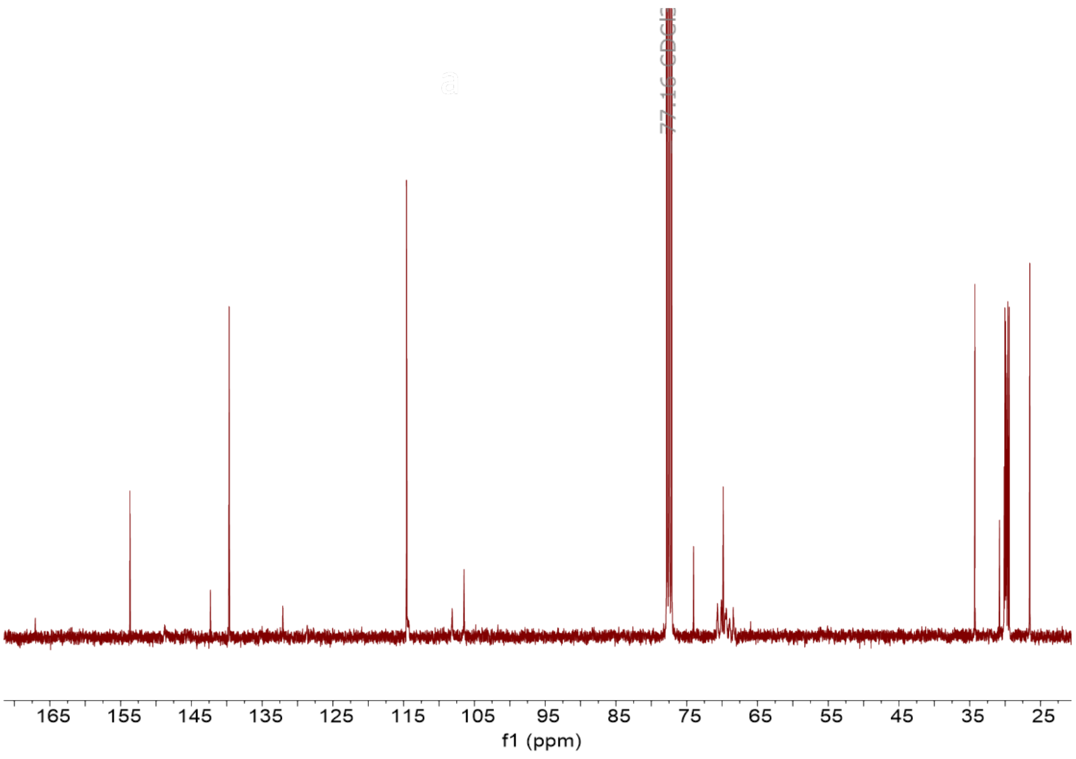

4. HSQC

4.1. HSQC compound 15C5BA

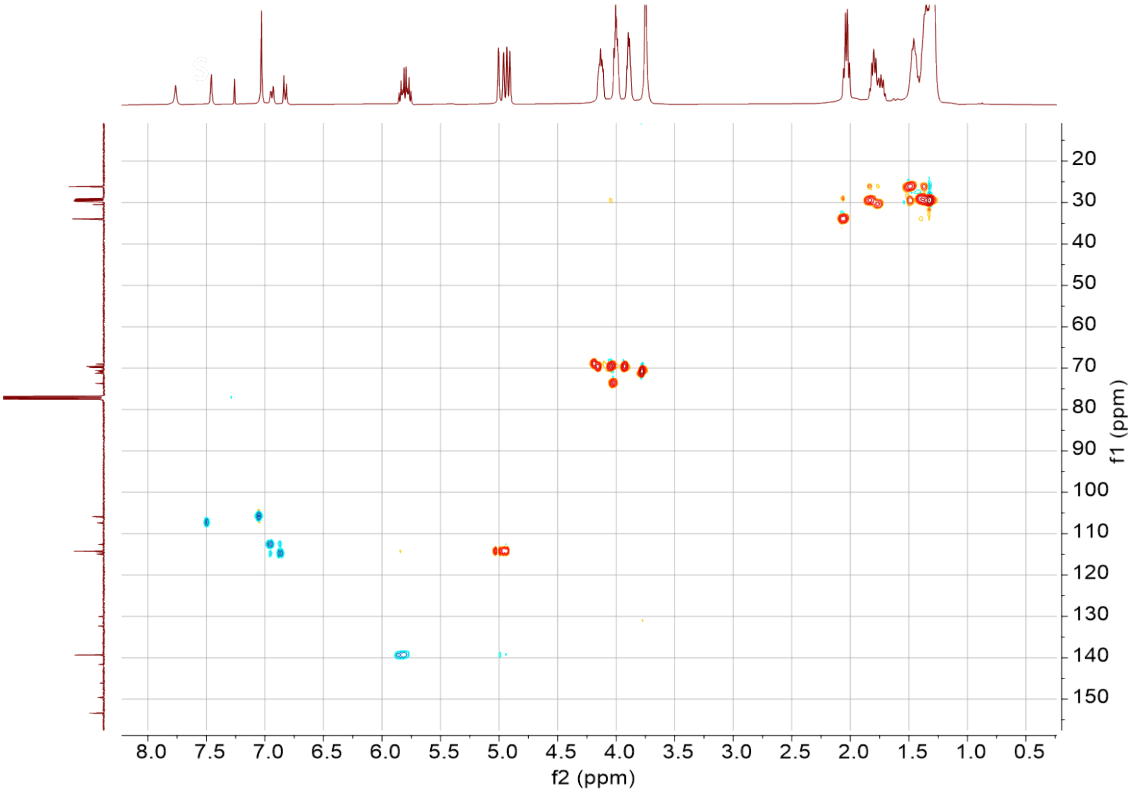

4.2. HSQC compound 15C5BA•NaI

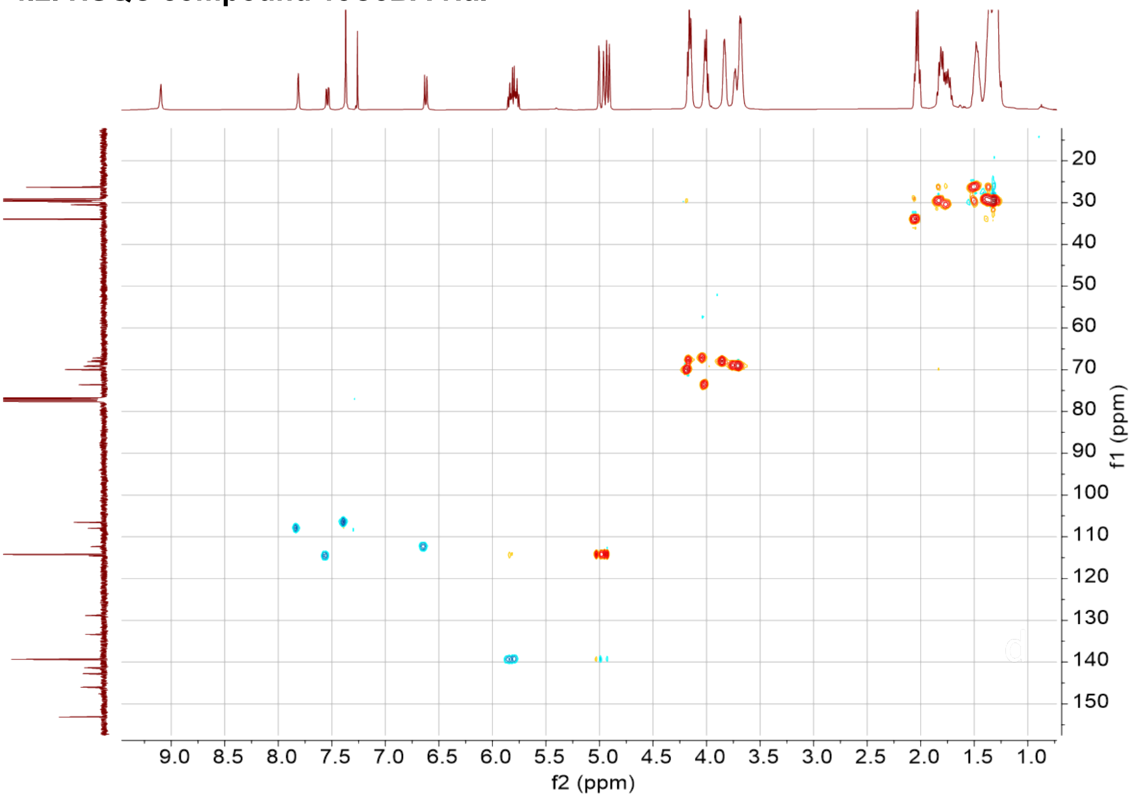

4.3. HSQC compound 15C5BA•NaSCN

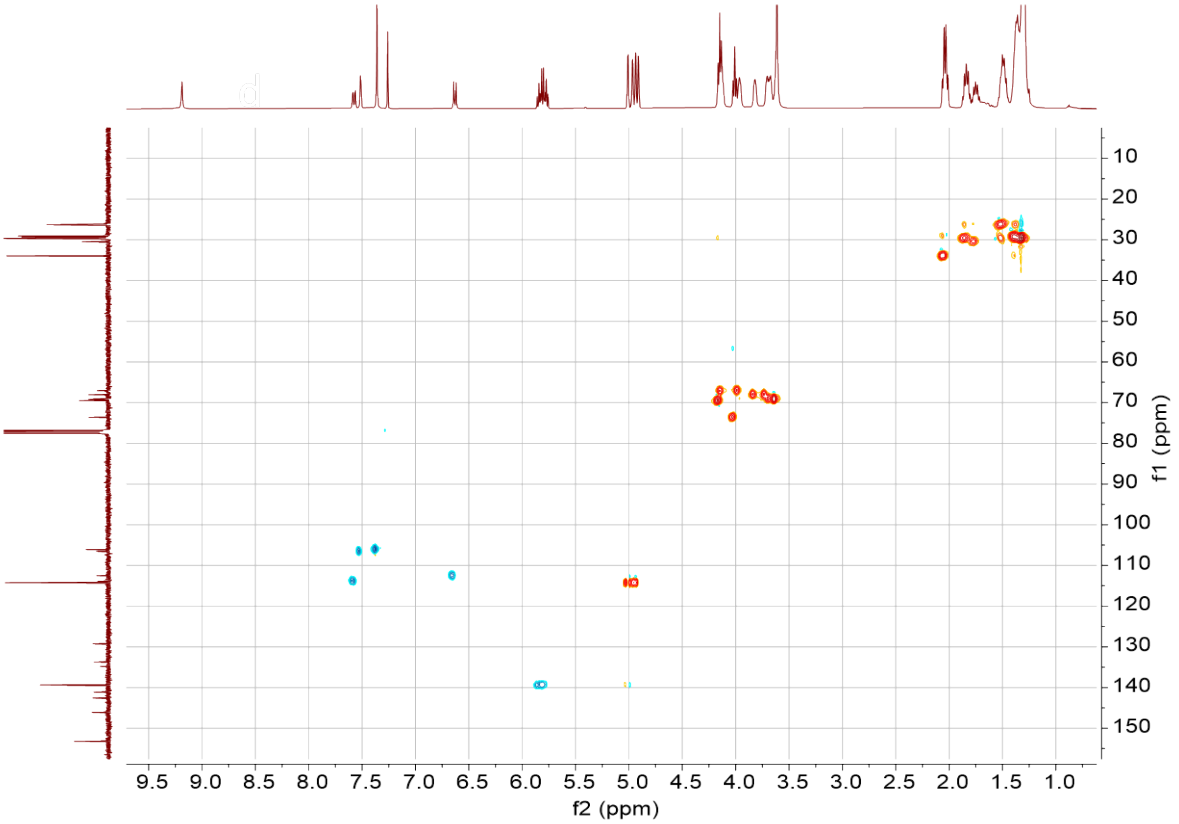

4.4. HSQC compound 15C5BA•$NaBF_4$

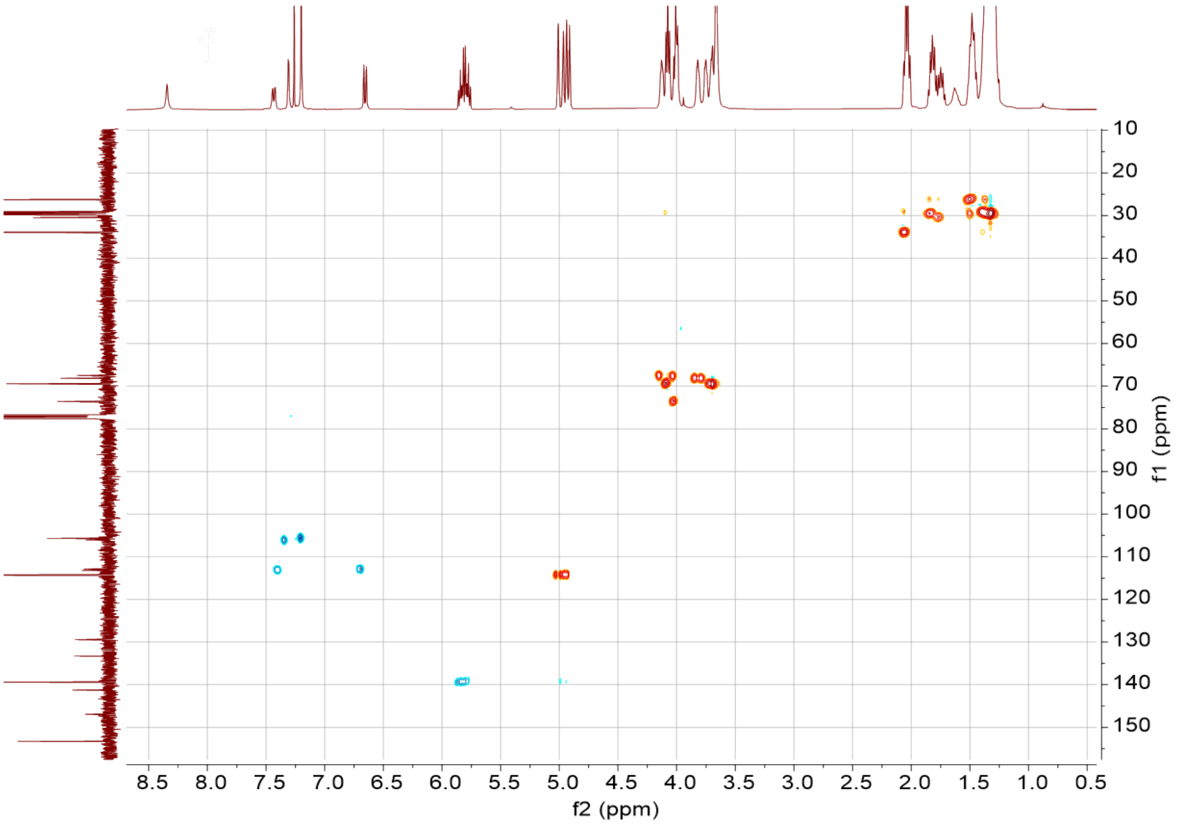

5. Differential Scanning Calorimetry

5.1. DSC thermogram of 15C5BA

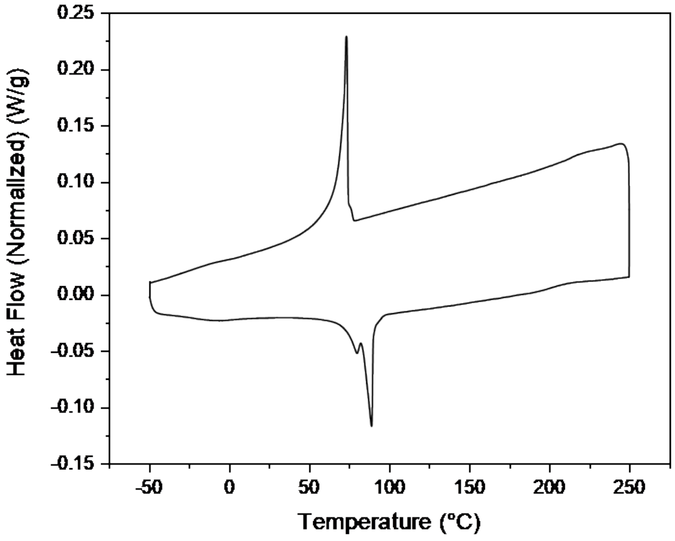

5.2. DSC thermogram of 15C5BA•NaI

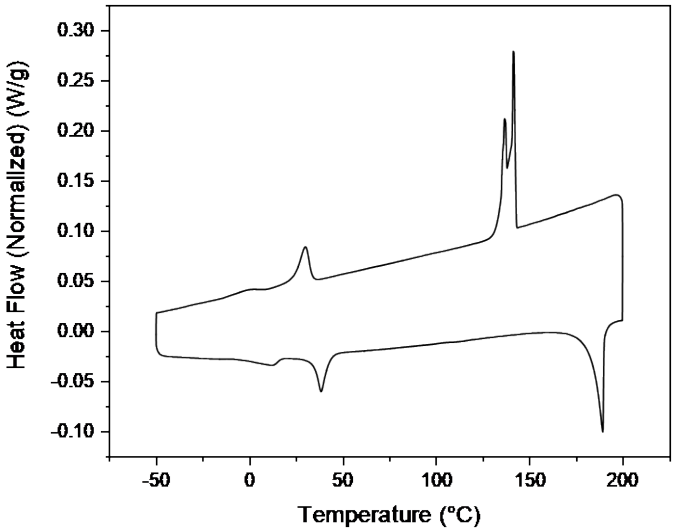

5.3. DSC thermogram of 15C5BA•NaSCN

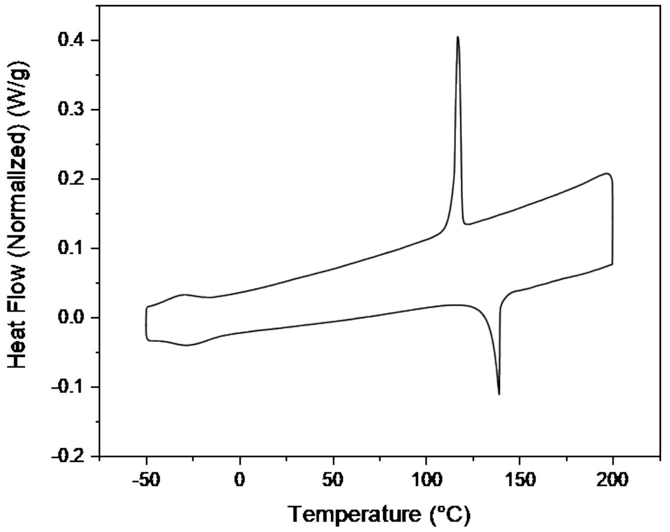

5.4. DSC thermogram of 15C5BA•$NaBF_4$

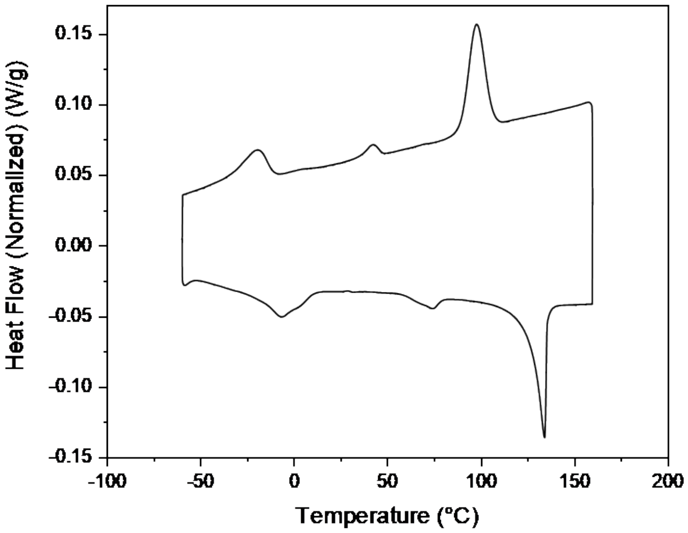

5.5. DSC thermogram of 15C5BA•$NaPF_6$

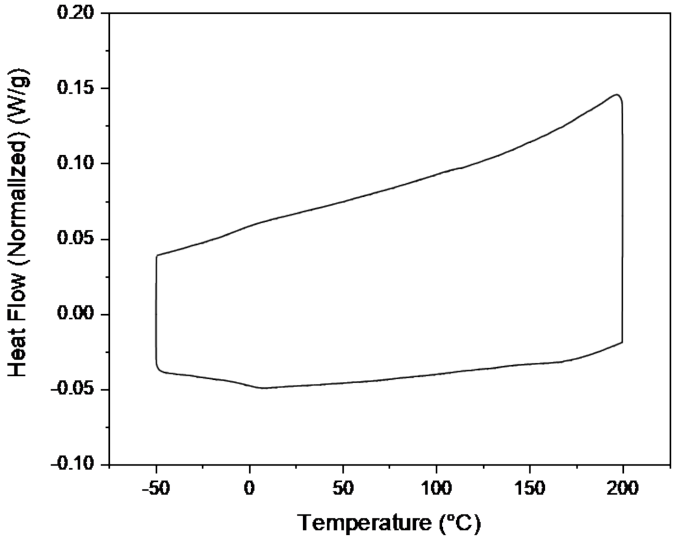

6. Polarized optical microscopy

6.1. POM of 15C5BA at different temperatures (5x magnification)

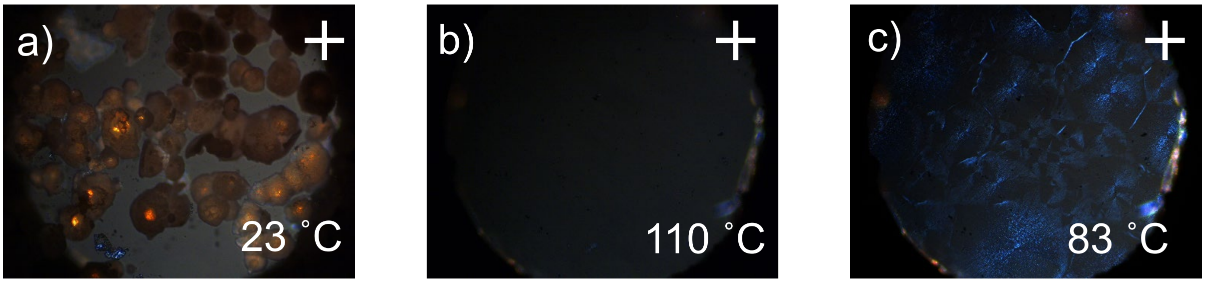

6.2. POM of 15C5BA•NaI at different temperatures (5x magnification)

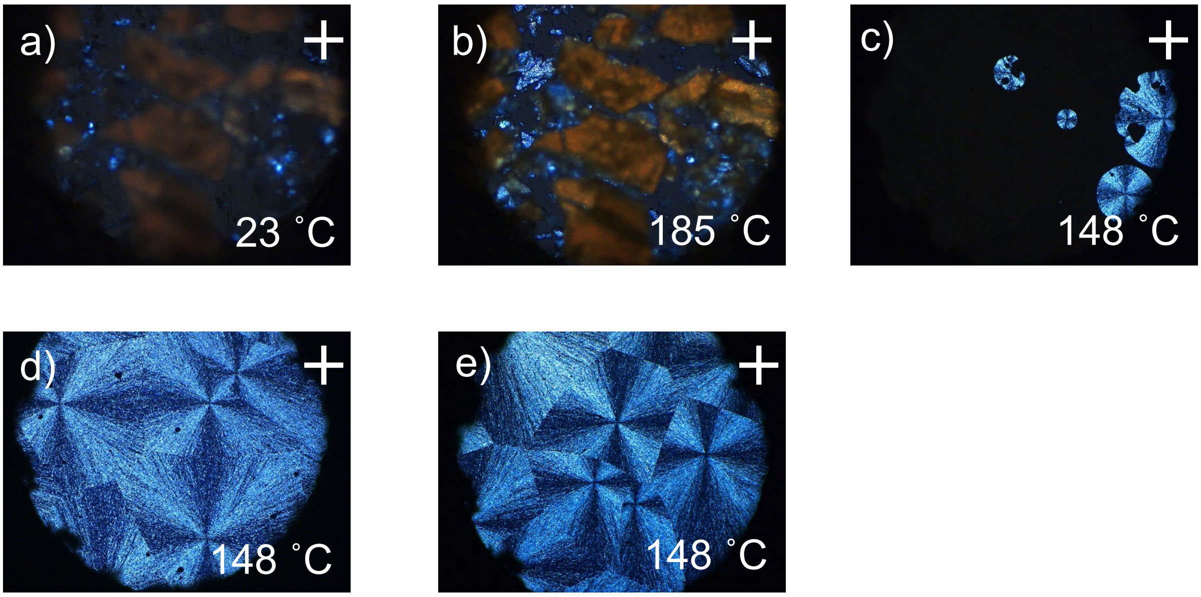

6.3. POM of 15C5BA•NaSCN at different temperatures (5x magnification)

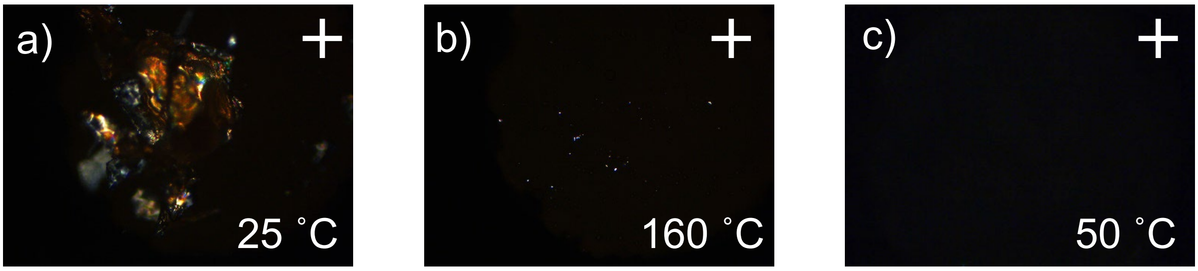

6.4. POM of 15C5BA•$NaBF_4$ at different temperatures (5x magnification)

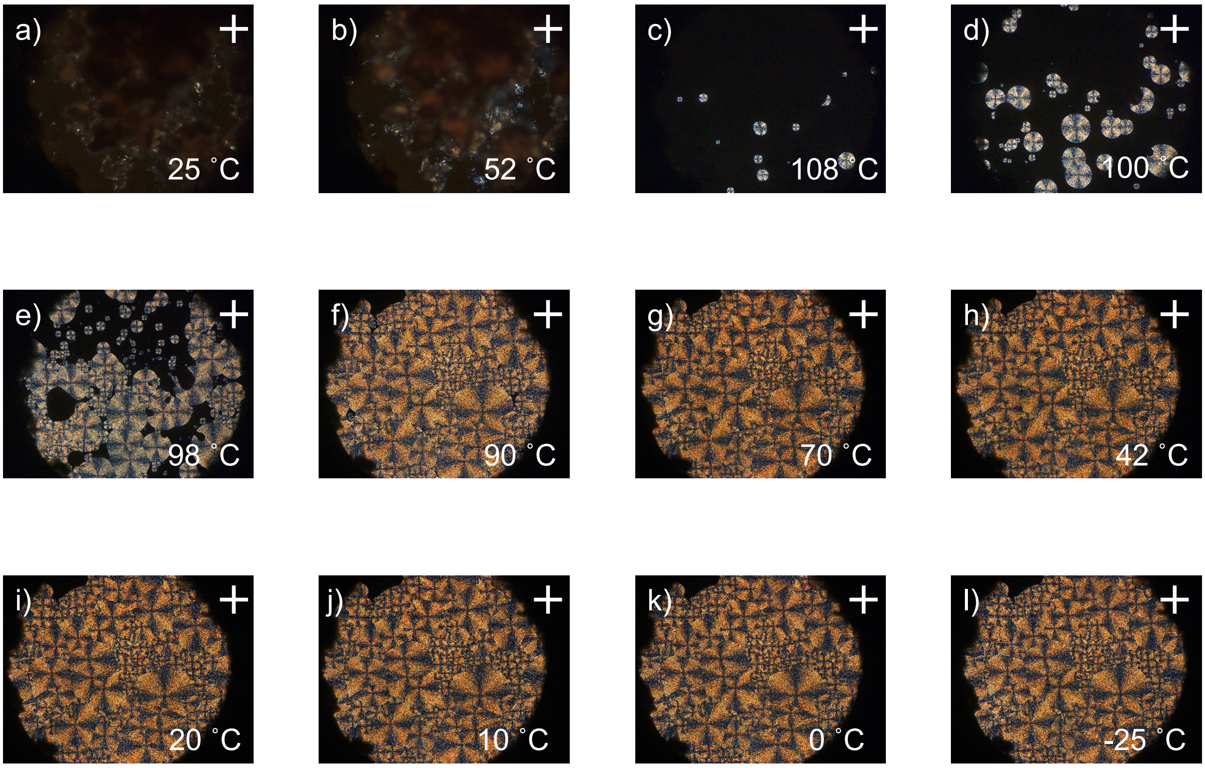

6.5. POM of 15C5BA•$NaPF_6$ at 93 °C (5x magnification)

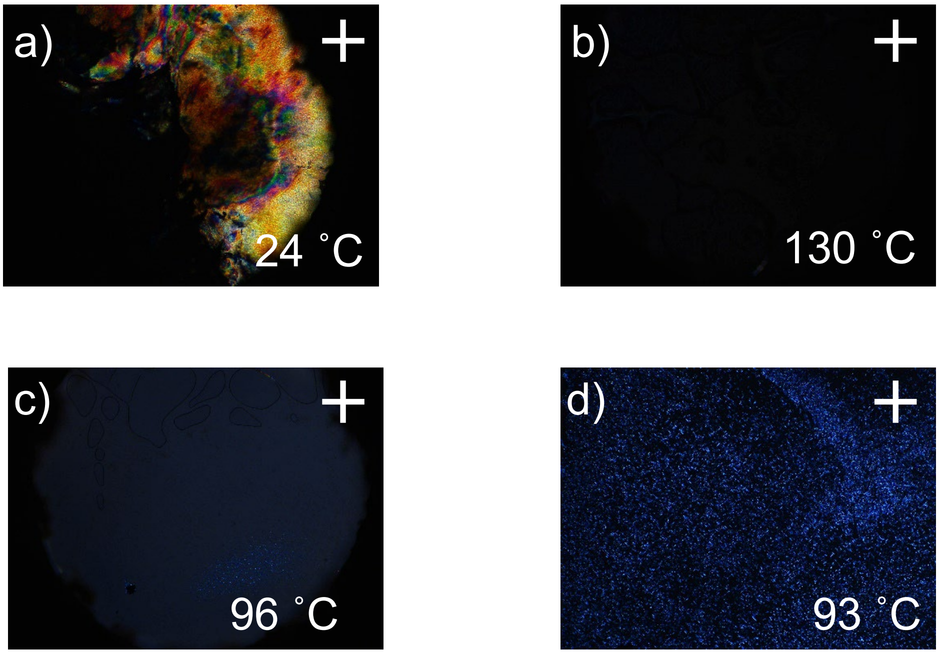

7. ATR FT-IR

7.1. ATR FT-IR of 15C5BA

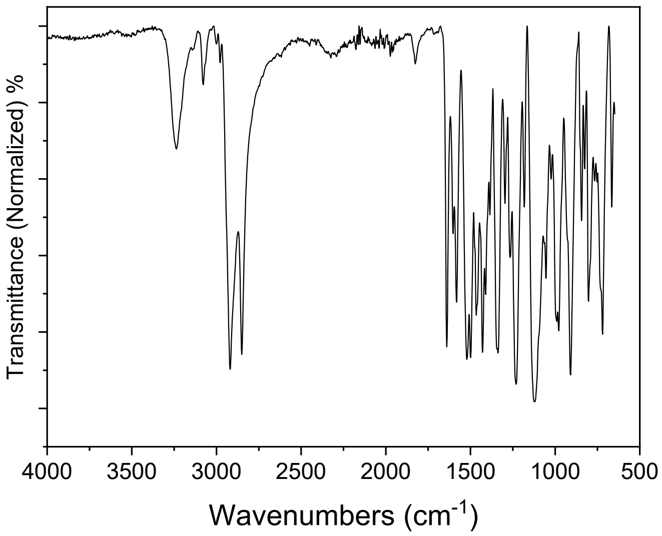

7.2. ATR FT-IT of 15C5BA•NaSCN

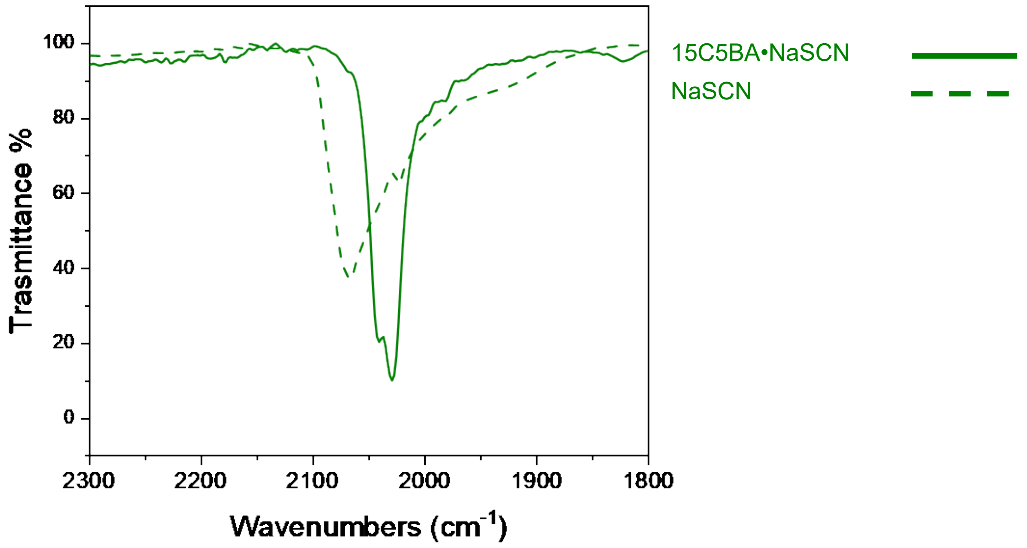

8. Medium- and wide- angle X-ray analysis

8.1. 1D and 2D diffractograms at different temperatures diffractograms and d values found and calculated for 15C5BA

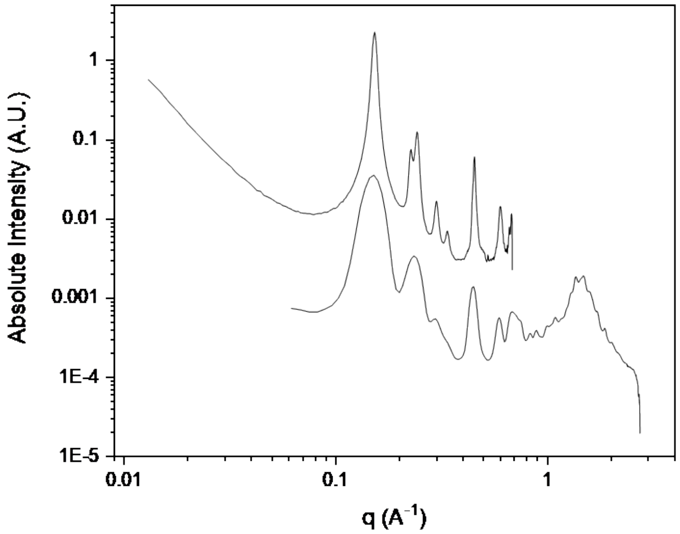

15C5BA		
d_{found} (Å)	$d_{calculated}$ (Å)	Peak assignment
40.99	41.75	d_100
27.58	27.60	d_010
25.81	25.56	d_110
20.90	20.87	d_200
18.55	18.58	d_210
13.82	13.92	d_020
10.44	10.44	d_400
9.18	9.29	d_030
8.33	8.35	d_500
6.97	6.96	d_040
6.20	6.19	d_630
4.54	4.53	d_060
3.60	-	d_001

8.2. 1D and 2D diffractograms at different temperatures diffractograms and d values found and calculated for 15C5BA•NaI

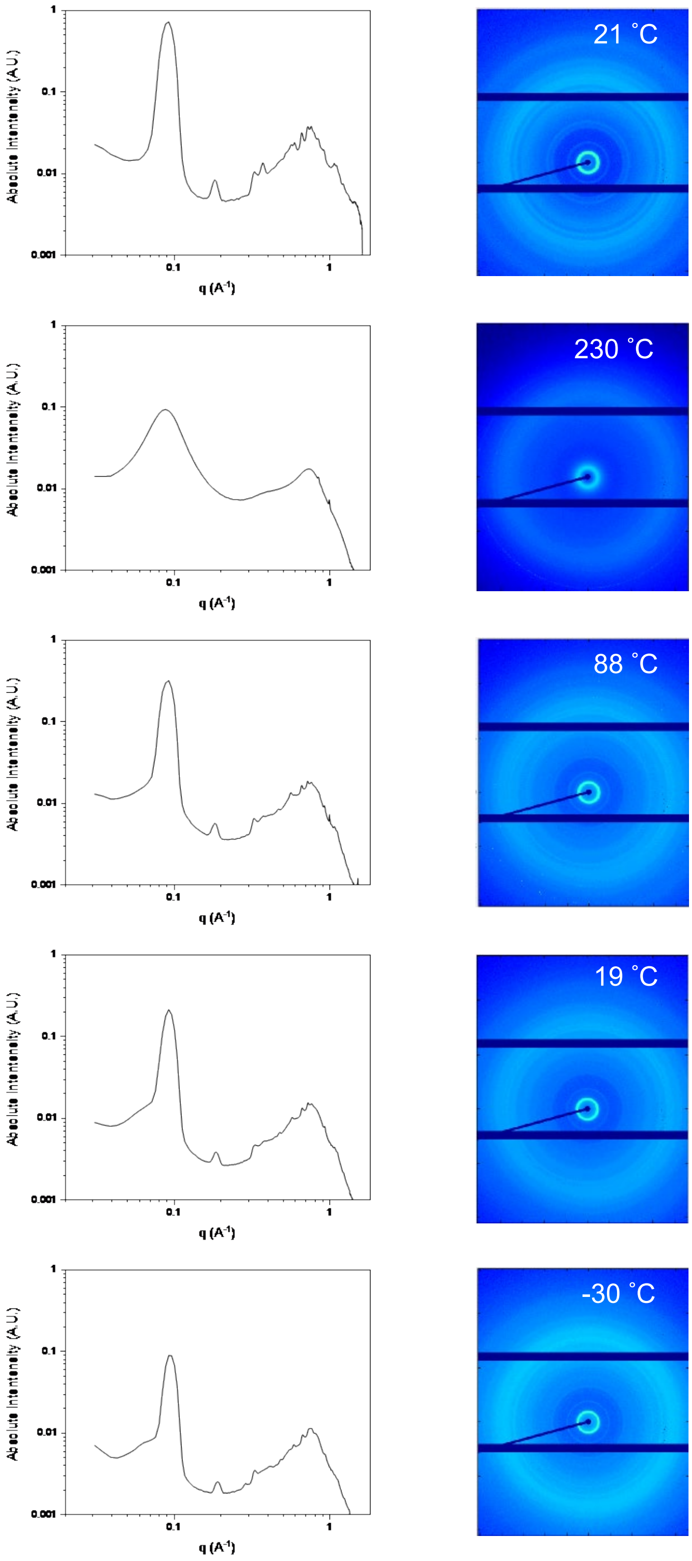

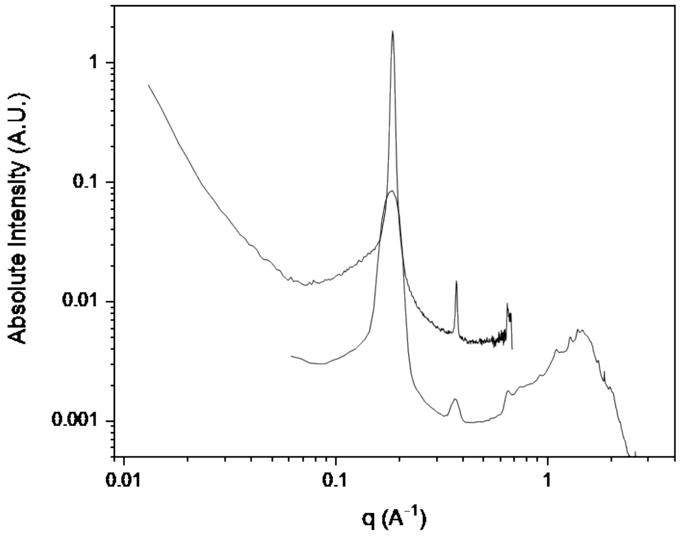

15C5BA•NaI at 88 ˚C		
d_{found} (Å)	$d_{calculated}$ (Å)	Peak assignment
br sh	38.23	d_010
33.74	33.66	d_100
16.83	16.83	d_200
9.56	9.56	d_040
8.30	8.41	d_400
6.77	6.73	d_500
5.68	5.61	d_600
4.88	4.81	d_700
4.28	4.21	d_800
3.59	-	d_001
3.36	3.37	d_(10)00

8.3. 1D and 2D diffractograms at different temperatures diffractograms and d values found and calculated for 15C5BA•NaSCN

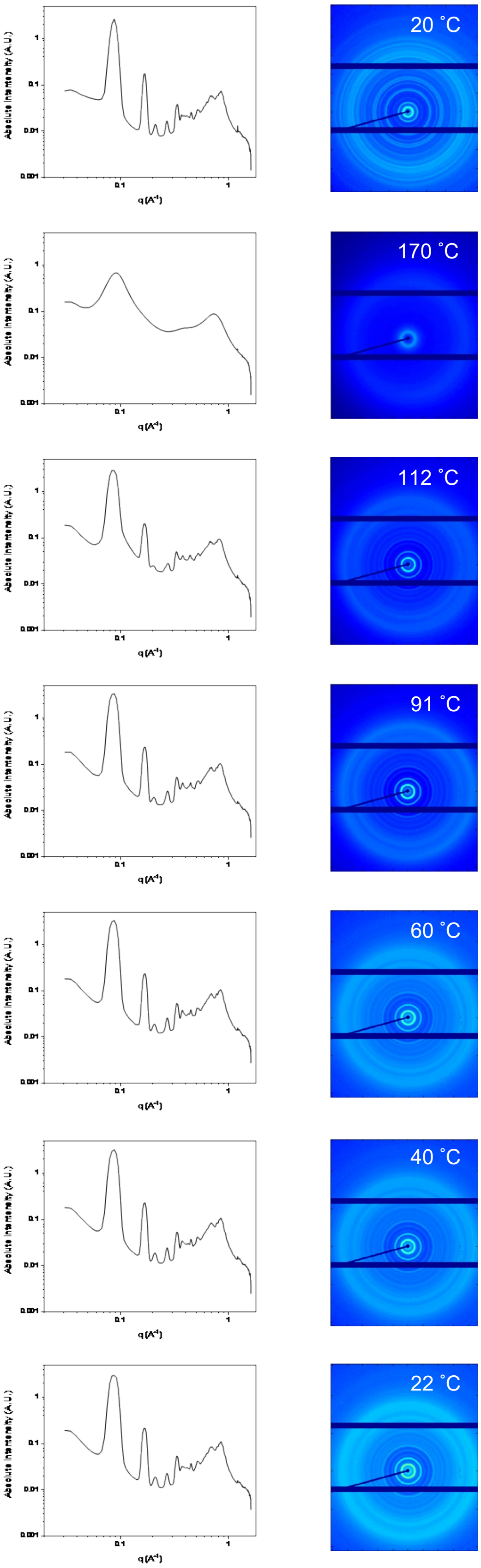

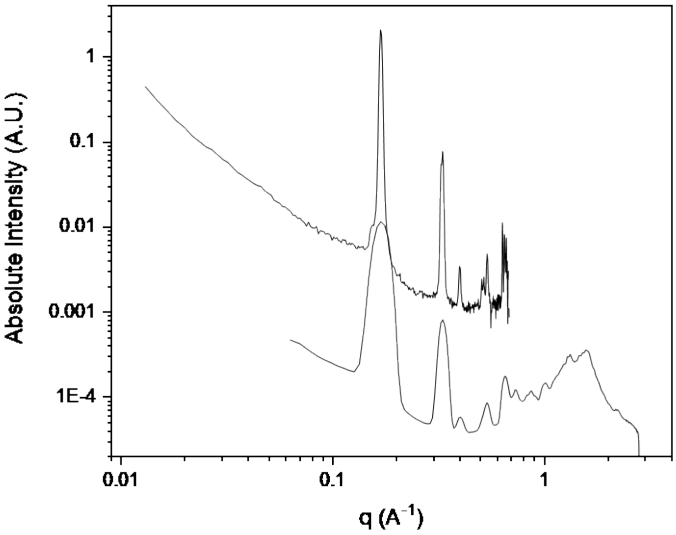

15C5BA•NaSCN at 60 ˚C		
d_{found} (Å)	$d_{calculated}$ (Å)	Peak assignment
37.38	37.15	d_100
18.69	18.57	d_200
19.08	19.11	d_020
15.62	15.44	d_120
12.16	12.74	d_300
12.00	12.08	d_220
11.39	11.10	d_130
9.76	9.58	d_230
9.56	9.56	d_040
9.29	9.29	d_400
7.28	7.43	d_500
6.16	6.19	d_600

8.4. 1D and 2D diffractograms at different temperatures diffractograms and d values found and calculated for 15C5BA•NaBF$_4$

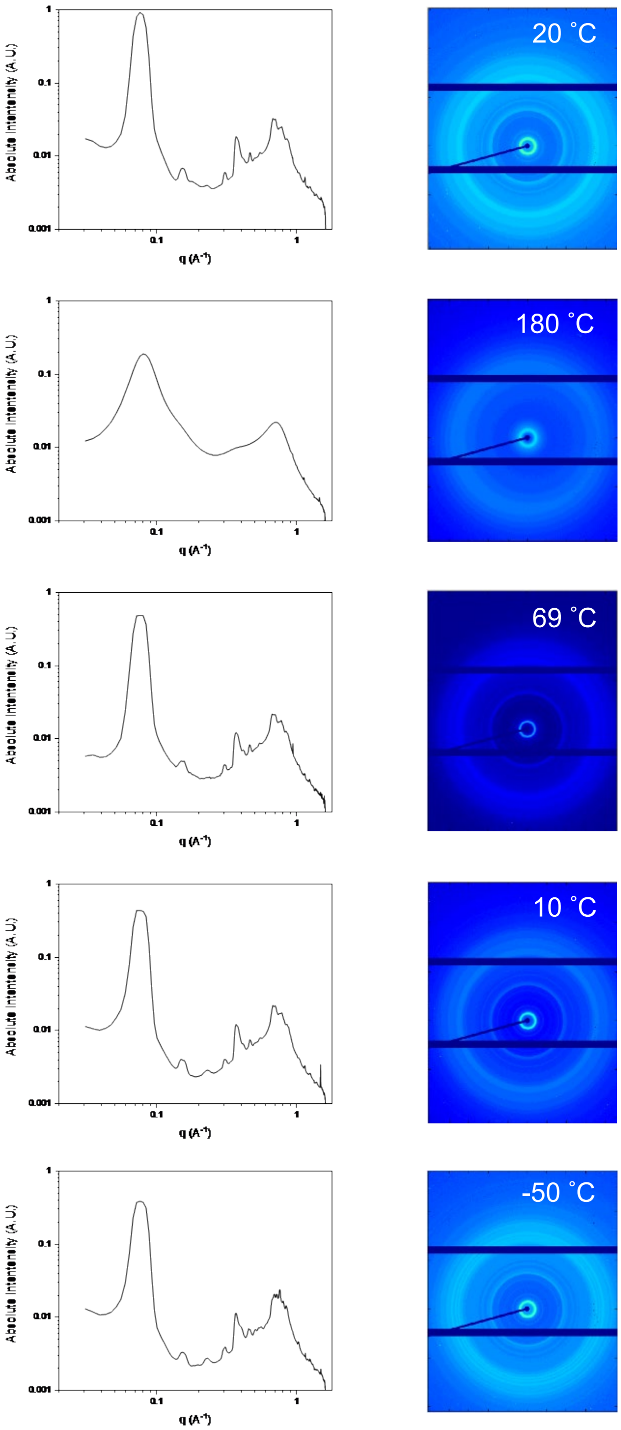

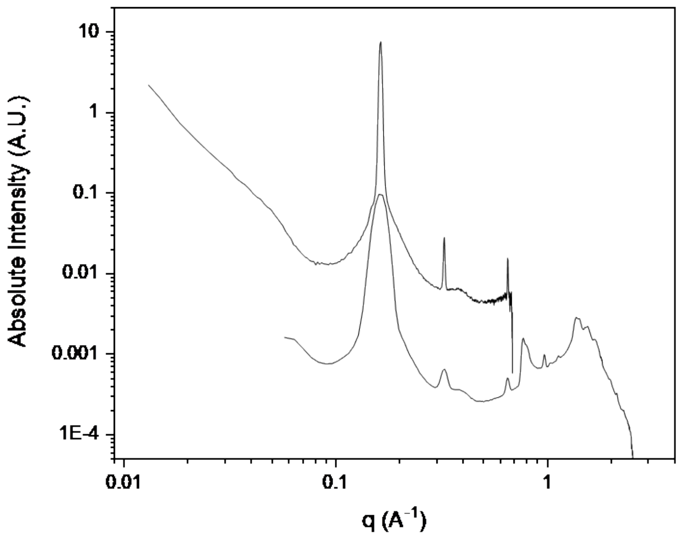

15C5BA•$NaBF_4$ at 69 °C		
d_{found} (Å)	$d_{calculated}$ (Å)	Peak assignment
39.15	39.15	d_100
19.52	19.57	d_200
16.50	16.50	d_010
13.00	13.05	d_300
9.74	9.79	d_400
8.26	8.25	d_020
7.92	7.83	d_500
6.54	6.52	d_600
3.81	-	d_001

8.5. 1D and 2D diffractograms at different temperatures and d values found and calculated for 15C5BA•$NaPF_6$

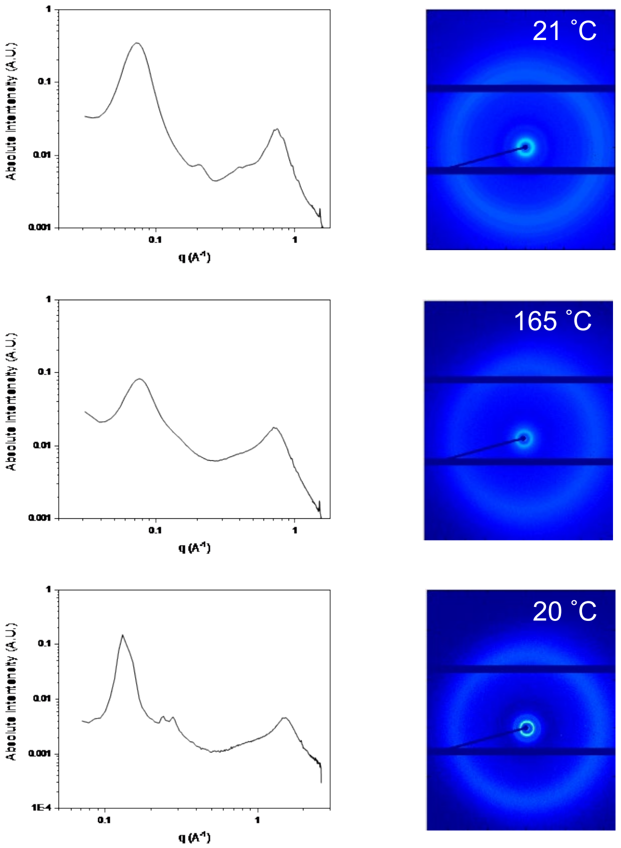

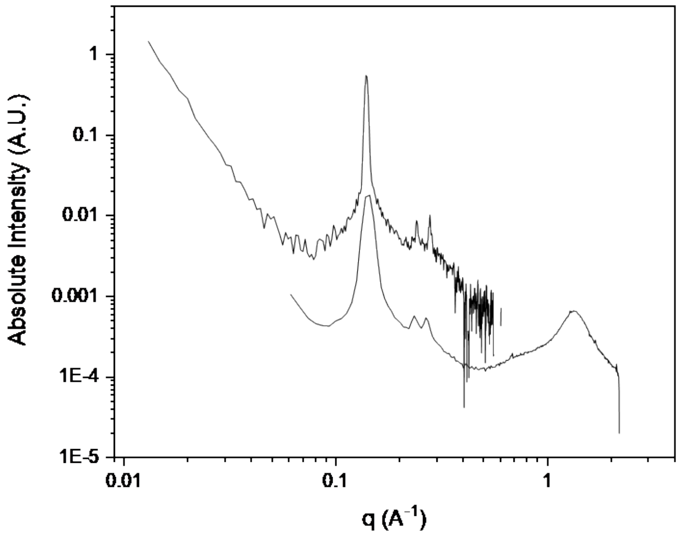

$15C5BA\cdot NaPF_6$ at 20 ˚C		
d_{found} (Å)	$d_{calculated}$ (Å)	Peak assignment
44.50	44.50	d_100
25.81	25.69	d_110
22.46	22.25	d_200
3.71	-	d_001

9. References:

[1] B. D. Cullity, *Elements of X-Ray Diffraction*, Addison-Wesley, **1978**.

[2] A. M. Van Herk, T. Dröge, *Macromol. Theory Simulations* **1997**, *6*, 1263–1276.
